# Ribosome maturation by the endoribonuclease YbeY stabilizes a type 3 secretion system transcript required for virulence of enterohemorrhagic *Escherichia coli*

**DOI:** 10.1074/jbc.RA117.000300

**Published:** 2018-04-20

**Authors:** Sean P. McAteer, Brandon M. Sy, Julia L. Wong, David Tollervey, David L. Gally, Jai J. Tree

**Affiliations:** From the ‡ Division of Infection and Immunity, The Roslin Institute, University of Edinburgh, Edinburgh EH25 9RG, Scotland, United Kingdom,; § School of Biotechnology and Biomolecular Sciences, University of New South Wales Sydney, Sydney 2033, Australia, and; ¶ Wellcome Trust Centre for Cell Biology, University of Edinburgh, Edinburgh EH9 3BF, Scotland, United Kingdom

**Keywords:** bacterial pathogenesis, gene regulation, RNA degradation, antibiotics, ribosomal RNA processing (rRNA processing), ribosome assembly, ribosomal ribonucleic acid (rRNA) (ribosomal RNA), precursor ribosomal RNA (pre-rRNA), ribonuclease, post-transcriptional regulation, anti-virulence, enterohemorrhagic E. coli, ketolide, small RNA, type 3 secretion, type III secretion, virulence factor, YbeY

## Abstract

Enterohemorrhagic *Escherichia coli* (EHEC) is a significant human pathogen that colonizes humans and its reservoir host, cattle. Colonization requires the expression of a type 3 secretion (T3S) system that injects a mixture of effector proteins into host cells to promote bacterial attachment and disease progression. The T3S system is tightly regulated by a complex network of transcriptional and post-transcriptional regulators. Using transposon mutagenesis, here we identified the *ybeZYX-Int* operon as being required for normal T3S levels. Deletion analyses localized the regulation to the endoribonuclease YbeY, previously linked to 16S rRNA maturation and small RNA (sRNA) function. Loss of *ybeY* in EHEC had pleiotropic effects on EHEC cells, including reduced motility and growth and cold sensitivity. Using UV cross-linking and RNA-Seq (CRAC) analysis, we identified YbeY-binding sites throughout the transcriptome and discovered specific binding of YbeY to the “neck” and “beak” regions of 16S rRNA but identified no significant association of YbeY with sRNA, suggesting that YbeY modulates T3S by depleting mature ribosomes. In *E. coli*, translation is strongly linked to mRNA stabilization, and subinhibitory concentrations of the translation-initiation inhibitor kasugamycin provoked rapid degradation of a polycistronic mRNA encoding needle filament and needle tip proteins of the T3S system. We conclude that T3S is particularly sensitive to depletion of initiating ribosomes, explaining the inhibition of T3S in the Δ*ybeY* strain. Accessory virulence transcripts may be preferentially degraded in cells with reduced translational capacity, potentially reflecting prioritization in protein production.

## Introduction

Enterohemorrhagic *Escherichia coli* (EHEC)^3^ causes outbreaks of severe foodborne diarrheal disease that may progress to potentially fatal hemolytic uremic syndrome caused by the release of Shiga toxins that damage Gb3-rich vascular endothelial cells, leading to thrombotic microangiopathy in the kidney and renal failure (1). Antibiotic treatment is contraindicated for EHEC infection as it may promote release of Shiga toxins, increasing the probability of developing hemolytic uremic syndrome (2). There is currently no commercially available treatment to inactivate Shiga toxins during EHEC infection, and patients predominately receive supportive care. Colonization of both the reservoir host (cattle) and infected humans occurs in the gastrointestinal tract and requires a type 3 secretion (T3S) system that enables the bacterium to inject a mixture of “effector” proteins into host cells that allow the bacterium to gain control of host cell processes and persist at the attachment site (3, 4). The T3S system is encoded within a pathogenicity island termed the locus of enterocyte effacement (LEE). The LEE is organized into five polycistronic operons that encode the basal apparatus (LEE1–3), needle filament and needle tip proteins (LEE4), and the adhesin intimin and its receptor Tir (LEE5) (5). The T3S system and secreted effectors are immunogenic and potentially only required during intimate attachment to host cells, so they are tightly regulated by a large number of transcriptional regulators in response to internal signals (GlrRA) and environmental cues (temperature, quorum sensing, and carbonate) and coordinated with other virulence factors (flagella, enterohemolysin, and Stx prophage) (6, 7). The LEE is also regulated post-transcriptionally by the small RNA chaperone Hfq (8–10), small RNAs (11), and the carbon storage regulator CsrA (12). Recent research has demonstrated that post-transcriptional regulation of the LEE in enteropathogenic *E. coli* by CsrA is antagonized by the LEE5-encoded effector chaperone CesT, establishing a post-transcriptional feedback loop that allows timed secretion of Tir and demonstrates the sophisticated level of post-transcriptional regulation that occurs during biogenesis of this complex molecular machine (13).

Active translation is known to stabilize mRNAs in both prokaryotes and eukaryotes (14–17). The effects of mutations in the ribosomal binding site that increase or decrease ribosome occupancy are correlated with altered mRNA stability (18–21). Mechanistically, elongating ribosomes appear to sterically shield the coding sequence from RNases (22), and initiating ribosomes may specifically prevent access by RNase E to the 5′-end of the transcript (23, 24). Conceptually, linking mRNA stability with translation may prevent aberrant transcripts from accumulating in the cell or facilitate rapid degradation of RNA fragments that do not contain appropriate translation signals (14).

Ribosome availability within the cell is influenced by the rate of maturation, a complex process that requires cleavage, modification, and temporal binding of a host of proteins to ribosomal RNAs. Recently, a critical step in maturation of the 16S rRNA 3′-end was shown to be mediated by the endoribonuclease YbeY, a highly conserved protein present in most bacteria. Deletion of *ybeY* leads to depletion of mature 70S polysomes in *E. coli*, *Vibrio cholerae*, and *Yersinia enterocolitica* as well as *Arabidopsis thaliana* chloroplasts (25–29). In addition to its role in 16S rRNA 3′-end maturation, YbeY also appears to monitor the integrity of mature 70S particles and, with the exoribonuclease RNase R, degrades defective 70S ribosomes *in vitro* (30).

Deletion of *ybeY* leads to highly pleiotropic phenotypes in growth rate, cell aggregation, sugar utilization, effector protein secretion, pigmentation, biofilm formation, toxin production, and mouse colonization as well as sensitivity to acid, temperature, antibiotics, hydroxyurea, hydrogen peroxide, and UV light (25, 31, 32). The highly diverse phenotypes manifested following *ybeY* deletion hint at a central role in gene regulation, and parallels have been drawn with the multiple phenotypes characterized in an *hfq* mutant (25, 26, 29, 31, 33). Indeed, small RNA abundance is altered in the Δ*ybeY* background, and YbeY possesses a MID domain with sequence and structural similarity to the microRNA-binding protein Argonaute from *Neurospora crassa* (29). In *Sinorhizobium meliloti*, these predictions were borne out by experiments showing coprecipitation of mRNAs, antisense small RNAs (sRNAs), and sRNAs with YbeY under native purification conditions (33). However, these analyses did not detect binding of YbeY to rRNA in *S. meliloti*, suggesting that its functions may have diverged from those seen in most bacteria and chloroplasts. A growing body of literature has now linked YbeY with small RNA function (25, 26, 29, 31, 33).

Using transposon mutagenesis of enterohemorrhagic *E. coli*, we identified YbeY as a regulator of type 3 secretion and motility. We found that YbeY does not directly bind transcripts encoding the T3S system, small RNAs, or mRNAs but contacts the 16S rRNA in a position consistent with maturation of the 16S rRNA 3′-end. Our results indicate that T3S is highly sensitive to depletion of initiating ribosomes due to destabilization of a T3S transcript, a fate potentially shared by many transcripts in the cell and resulting in highly pleiotropic phenotypes in *ybeY* mutants.

## Results

### Regulators of type 3 secretion in enterohemorrhagic E. coli identified by transposon mutagenesis

To identify novel regulators of T3S in EHEC, a chromosomal, translational fusion was constructed between the T3S effector protein Tir and LacZ (*tir-lacZ*) in *E. coli* O157:H7 ZAP193 by allelic exchange replacing *lacZYA* (Table S1). Colonies of this strain were blue on M9-glycerol agar plates supplemented with 50 μg·ml^−1^ X-Gal (M9GX). A library of random transposon Tn5 insertions was produced in this strain using the λpir method (34) with S17–1/pUT-miniTn5Km2 as a donor. Conjugation was carried out in lysogeny broth, and transposon insertions were selected on M9GX supplemented with kanamycin and nalidixic acid. Colonies with reduced blue color, indicating decreased expression, were selected.

**Figure 1.**
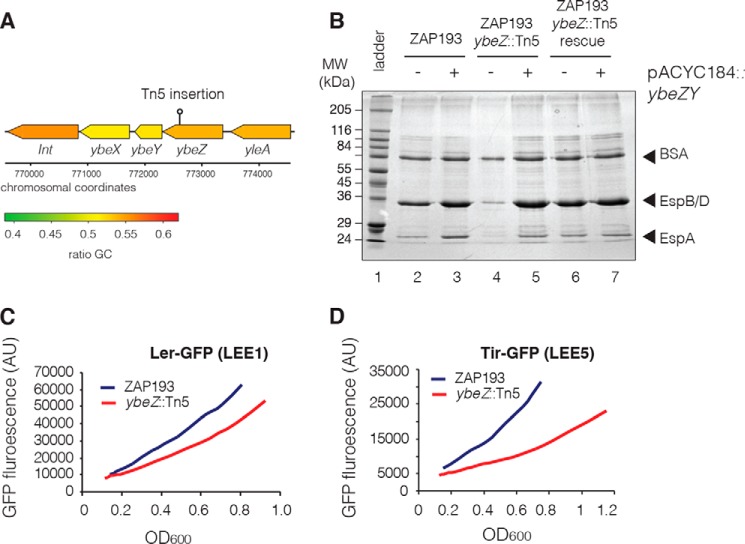
**A transposon insertion in *ybeZ* reduces T3S in enterohemorrhagic *E. coli* O157:H7 str. ZAP193.** *A*, graphical representation of the *ybeZYX-Int* operon and position of the Tn5 transposon insertion. *B*, Coomassie-stained T3S profile of secreted proteins for ZAP193, isogenic *ybeZ::*Tn5, and Tn5 mutant repaired by marker rescue. Each strain was also complemented in *trans* with *ybeZY* (indicated as +/− *above lane*). *Arrows* indicate abundant proteins in the secreted profile. BSA is used as a coprecipitant and loading control. *C*, expression from LEE1 using a Ler-GFP translational fusion in WT (ZAP193) and the isogenic *ybeZ::*Tn5 strains. *D*, expression from LEE5 using a Tir-GFP translational fusion in WT (ZAP193) and the isogenic *ybeZ::*Tn5 strains. *AU*, arbitrary units.

Insertion sites were mapped as described previously (35). Notably, insertions were recovered within the LEE, including the known T3S regulator *grlA*, confirming that our approach recovers *bona fide* T3S regulators. We confirmed that the altered Tir-LacZ expression was due to the mapped Tn5 insertion using marker rescue. In the case of the uncharacterized gene *ybeZ* (Fig. 1*A*), this restored LacZ expression to WT levels. However, the apparent phenotypes of the other novel genes tested were not restored. These may carry secondary mutations and were not studied further. The *ybeZ::*Tn5 strain was tested for effects on the expression of other T3S proteins by SDS-PAGE analysis of secreted proteins (Fig. 1*B*). The *ybeZ::*Tn5 strain showed reduced levels of T3S needle tip proteins EspB/D and filament protein EspA (Fig. 1*B*, *lane 4*) that could be restored by ectopic expression of the *ybeZY* operon (Fig. 1*B*, *lane 5*) or Tn5 marker rescue (Fig. 1*B*, *lane 6*). This demonstrated that the effects of *ybeZ::*Tn5 are not specific to the fusion protein.

T3S expression is controlled by the master regulator Ler, which drives transcription of other LEE operons (5, 6). Constructs expressing translational fusions of Ler-GFP expressed from LEE1 (pAJR71) and Tir-GFP expressed from LEE5 (pAJR75) (36) were transformed into WT (ZAP193) and *ybeZ::*Tn5 strains and assayed for fluorescence under T3S-inducing conditions (Fig. 1, *C* and *D*). GFP expression was reduced for both pAJR71 and pAJR75, demonstrating that the *ybeZ* insertion does not act specifically at LEE5 and likely affected all LEE transcripts.

### YbeY is required for type 3 secretion in EHEC

**Figure 2.**
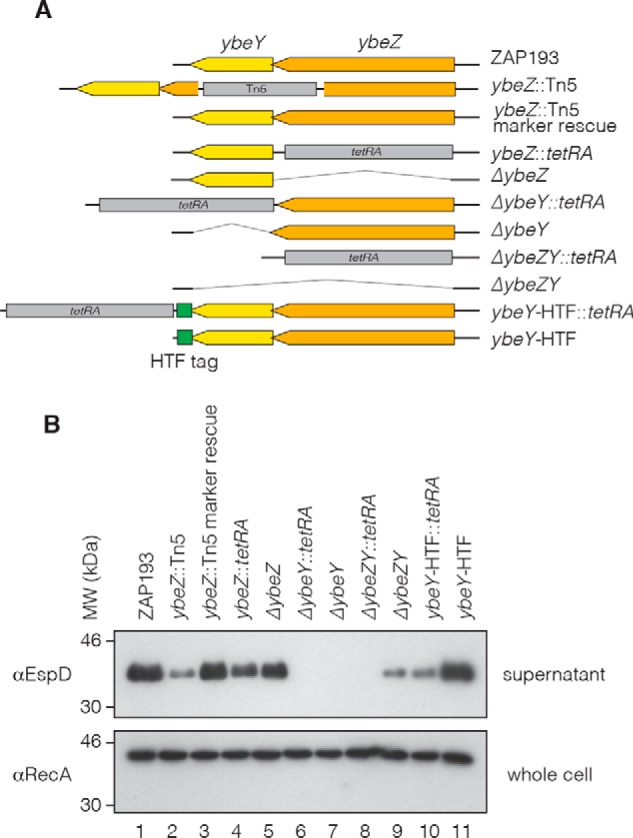
**YbeY is required for T3S and is disrupted by a polar *ybeZ::*Tn5 insertion.** *A*, graphical representations of the insertion and deletion genotypes (*tetRA*, Tn5, and *HTF* insertions are not to scale). *B*, Western blotting of secreted T3S system needle tip protein EspD in *ybeZ* and *ybeY* deletion and insertion backgrounds (genotype indicated *above lane*). RecA from the whole cell fraction was used as a loading control for each lane.

Unusually for *E. coli*, the stop codon of *ybeZ* overlaps the start codon of the downstream *ybeY* coding sequence. To determine which gene is required for T3S, the *ybeZ::*Tn5 insertion was complemented in *trans* using a pACYC184 construct containing the *ybeZ* promoter and coding sequence without *ybeY.* In contrast to the results obtained by expression of both *ybeZ* and *ybeY* (Fig. 1*B*, *lanes 4* and *5*), WT expression was not restored using *ybeZ* alone (data not shown). These data indicated that the *ybeZ::*Tn5 insertion is polar and that the downstream *ybeY* gene contributes to the T3S phenotype. To better distinguish the roles of YbeZ and YbeY, we constructed a series of insertions and clean deletions in both genes (Fig. 2*A*). Type 3 secretion was assayed by Western blotting for the needle tip protein EspD in culture supernatants, and RecA in whole cell fractions was used as a loading control (Fig. 2*B*). Replacing *ybeZ* with a tetracycline resistance cassette (*tetRA*) while retaining the *ybeY* start codon reduced expression of the T3S protein EspD (Fig. 2*B*, *lane 4*), in line with the original transposon insertion. Excision of the *tetRA* cassette (*ybeZ* clean deletion) restored WT T3S (Fig. 2*B*, *lane 5*), confirming that the insertion is polar. Replacing *ybeY* with the *tetRA* cassette (and retaining the *ybeZ* stop codon) completely abolished T3S and was not restored to WT by removing the *tetRA* cassette (Fig. 2*B*, *lanes 6* and *7*). This demonstrated that *ybeY* is responsible for the T3S phenotype.

Replacing both *ybeZ* and *ybeY* with the *tetRA* cassette also abolished T3S, but this was partly restored by removal of the cassette (Fig. 2*B*, *lanes 8* and *9*). This indicates that the *ybeZY::tetRA* insertion has additional polar effects on genes further downstream. The repressive effect seen in the *ybeY* clean deletion may also be partially dependent on YbeZ. This conclusion is supported by the observation that inserting *tetRA* after the *ybeY* coding sequence (to introduce affinity tags into YbeY for UV cross-linking and analysis of cDNAs (CRAC)) reduced T3S, and WT expression could be restored by removing the cassette (Fig. 2*B*, *lanes 10* and *11*). These results demonstrate that YbeY is required for T3S but also indicate that an interplay between YbeY-YbeZ and downstream genes may exacerbate the T3S phenotype in the *ybeY* deletion.

### A ybeY mutant in enterohemorrhagic E. coli has defective 16S rRNA maturation, cold sensitivity, and reduced motility

**Figure 3.**
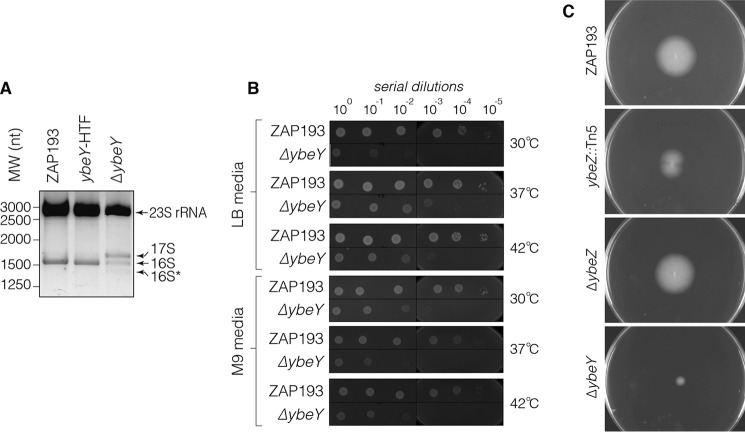
**Deletion of YbeY leads to defective 16S rRNA maturation, cold sensitivity, and reduced motility.** *A*, total RNA was extracted from WT ZAP193, *ybeY*-HTF strain, and *ybeY* after a 1-h temperature shift from 37 to 45 °C and separated on a 1.5% MetaPhor agarose gel. The accumulation of 17S and 16S* rRNA species is indicated. *B*, WT ZAP193 and the isogenic Δ*ybeY* strains were cultured on LB medium or M9-glycerol (indicated on the *right*) at 37, 30, or 42 °C as indicated. The WT and Δ*ybeY* strains were serially diluted and spotted onto plates for overnight growth. *C*, the motility of ZAP193, isogenic *ybeZ::*Tn5 insertion, and *ybeZ* and *ybeY* clean deletions was assessed using motility agar. Migration from the center of the plate indicates the relative motility of each strain.

YbeY is a highly conserved endoribonuclease that is required for maturation of the 3′-end of 16S rRNA (30). Deletion of *ybeY* in EHEC resulted in a 16S rRNA maturation defect similar to that reported for *E. coli* K12. However, accumulation of 17S pre-rRNA and 16S* in *ybeY* EHEC was observed only after shifting the culture from 37 to 45 °C for 1 h (Fig. 3*A*). This suggests that ribosome maturation in the EHEC *ybeY* mutant is more heat-tolerant than in *E. coli* K12.

YbeY mutants have significantly reduced mature 70S ribosomes and exhibit pleiotropic phenotypes in a range of bacteria, including *E. coli*, *Y. enterocolitica*, and *V. cholerae* (23, 24, 26). To determine whether deleting *ybeY* in EHEC has similar pleiotropic affects that extend beyond T3S, we tested temperature sensitivity, a common phenotype for mutations that disrupt ribosome biogenesis, and previously observed for a *ybeY* mutation in commensal *E. coli* (28). Deleting *ybeY* in EHEC rendered the cells sensitive to low-temperature growth (30 °C) on LB (Fig. 3*B*). The *ybeY* mutant grew poorly on M9-glycerol plates (Fig. 3*B*), and incubation at 30 or 42 °C did not decrease growth further.

The T3S system is evolutionarily related to flagella that are similarly encoded on multiple large polycistronic operons. We looked to determine whether motility was also reduced in our *ybeY* mutant (Fig. 3*C*). The *ybeY* clean deletion, but not the *ybeZ* clean deletion, had significantly reduced motility, indicating that expression of flagella is also reduced in the absence of YbeY. Our results demonstrate that deletion of *ybeY* reduces expression of T3S, reduces motility, and, consistent with known ribosome maturation defects, renders growth of EHEC sensitive to low temperatures.

### YbeY does not bind mRNAs within the LEE or regulatory small RNAs

**Figure 4.**
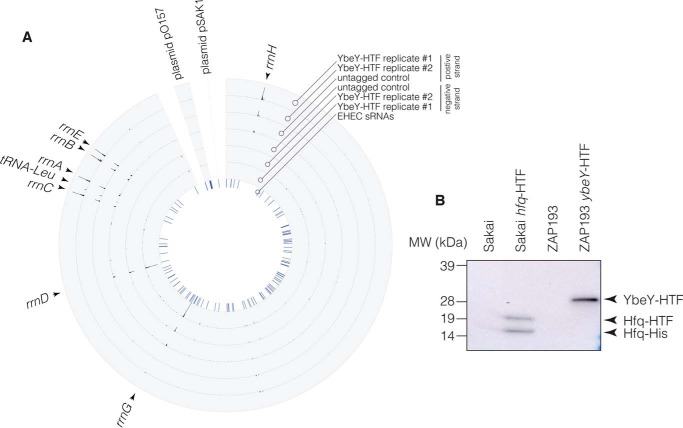
**YbeY binds ribosomal RNAs and is not associated with regulatory small RNA.** *A*, CRAC was used to identify RNAs interacting with YbeY *in vivo*. The YbeY genotype for each strain is indicated (*right*) where YbeY-HTF are the experimental replicates, and the untagged strain is the negative control. The *outer rings* represent the YbeY binding profile from the positive strand of the transcriptome, and the *inner rings* represent the binding to the negative strand of the transcriptome. The positions of small RNAs are indicated in the *innermost ring* (*blue*). The positions of the seven copies of rRNA (*rrnA–H*) are indicated by *arrows* on the *outer edge* of the plot. *B*, the YbeY-HTF complex purifies at levels comparable with Hfq-HTF. Western blot analysis of YbeY-His and Hfq-His purification using M2 anti-FLAG resin is shown. Proteins were extracted from equal amounts of biomass, electrophoresed, and blotted with anti-His antibody.

Recent publications have noted that the pleiotropic phenotypes of *ybeY* mutants are similar to defects seen in *hfq* mutants (25, 26, 29, 31, 33). These similarities led to suggestions that YbeY might function in sRNA-dependent regulation of mRNA translation or stability, although direct evidence for YbeY interactions with sRNAs is limited. To determine whether YbeY is directly associated with sRNAs, we used the CRAC technique, which allows high-resolution mapping of *in vivo* RNA–protein interactions transcriptome-wide (37, 38). To facilitate YbeY CRAC, we C-terminally tagged the chromosomal copy of YbeY with a dual His_6_-3xFLAG affinity purification tag (38). We confirmed that C-terminally tagged YbeY is functional as assessed by EspD expression and 16S rRNA maturation (Figs. 2*A*, *lane 11*, and 3*A*). The YbeY-HTF fusion and WT (ZAP193) strains were cultured under T3S-inducing condition (supplemented minimal essential medium (MEM)-HEPES) and UV cross-linked while actively growing as described previously (37). YbeY-RNA complexes were purified over M2 anti-FLAG resin, eluted by tobacco etch virus (TEV) protease cleavage of the FLAG tag, and subsequently purified over nickel-nitrilotriacetic acid (NTA) resin under denaturing conditions. Linkers were ligated during purification, and cDNA libraries were synthesized from eluted RNA, PCR-amplified, and sequenced using the Illumina MiSeq platform. The YbeY binding profile was analyzed using the pyCRAC package (39), and statistically significant peaks were identified using pyCalculateFDR as described previously (40) (Fig. 4*A*). The seven rRNA loci cannot be distinguished in short-read sequences, so all but one of the rRNA operons (*rrnC*) was masked, and YbeY-bound reads were remapped to the genome for specific analysis of rRNA transcripts. We identified nine statistically significant YbeY-binding sites that were identified in both YbeY-HTF experimental replicates and absent from our untagged control. Eight binding sites were positioned within the ribosomal rRNA (Fig. 4*A*). The remaining site was recovered in tRNA^Leu^. No binding sites were recovered within regulatory sRNAs. These results are in contrast to CRAC of Hfq and RNase E, which recovered many sRNAs (37, 38). Notably, our dual-affinity tag purification protocol recovers comparable amounts of YbeY and Hfq (Fig. 4*B*), indicating that lower protein recovery is not responsible for loss of sRNA or mRNA signal. We conclude that the major RNA targets for YbeY are the ribosomal RNAs.

### YbeY binds 16S rRNA adjacent to Era and the 3′-end

**Figure 5.**
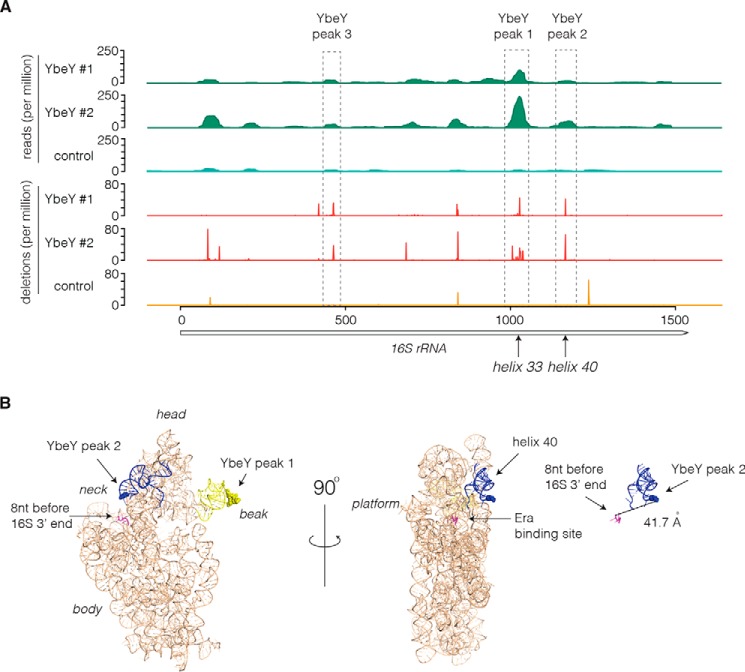
**YbeY binds 16S rRNA in close proximity to the 3′-end and Era-binding site.** *A*, YbeY UV cross-linking sites with 16S rRNA (*rrnC*). Sequencing reads recovered (*green*) and contact-dependent sequence deletions (*red*/*orange*) are indicated for HTF-tagged YbeY and untagged controls. The *dashed boxes* indicate the position of reproducible deletions within 16S rRNA. *B*, structure of 16S rRNA with the 30S subunit. YbeY-binding sites 1 (*yellow*) and 2 (*blue*) are indicated, and contact-dependent deletions are shown as *filled balls*. The 3′-end of the 16S rRNA structure is shown in *pink* (the mature 16S rRNA end is 8 nt after the 3′-end shown). The approximate Era-binding site is indicated (*middle*), and the distance between YbeY-induced deletions and the 16S rRNA structure 3′-end is shown (*right panel*).

YbeY was proposed to mature the 3′-end of 16S rRNA by endonucleolytic cleavage of the 17S pre-rRNA (30), although this conclusion has been questioned (41). Five statistically significant peaks that were present in both experimental replicates and absent from the control were identified with the 16S rRNA sequence. Reproducible sites of sequence deletions, which indicate precise protein-nucleotide cross-links, were identified at nucleotide positions 1028 (YbeY-binding site 1 in helix 33) and 1167 (YbeY peak 2 in helix 40), and a minor site was identified at nucleotide 464 (YbeY peak 3) but not in the control sample (Fig. 5*A*). Mapping these binding sites onto the 16S rRNA crystal structure (Protein Data Bank code 3OFA; Fig. 5*B*) showed that peak 1 is located in the “beak” of 16S rRNA that is positioned on the opposite side of 16S rRNA to the mature 3′-end. Peak 2 is positioned within helix 40 at the “neck” of the 16S rRNA structure. The 30S ribosome structure lacks the 3′-terminal 8 nt of the 16S rRNA. Mapping sequence deletions within binding site 2 positions YbeY 41.7 Å from the 3′-end of the visible 16S rRNA structure. This would be consistent with 3′ cleavage of 16S rRNA by YbeY bound at site 2.

Bacterial two-hybrid assays indicate that YbeY interacts with the GTPase Era, which is also required for 16S rRNA maturation (42). Cryo-EM of Era in complex with the 30S subunit demonstrate that Era binds at the neck between the head and the platform (Fig. 5*B*) (43). This position is in good agreement with binding site 2 and would potentially position YbeY above Era in the pre-30S structure.

### The LEE4 transcript is destabilized in a ybeY mutant and phenocopied by a translation initiation inhibitor

**Figure 6.**
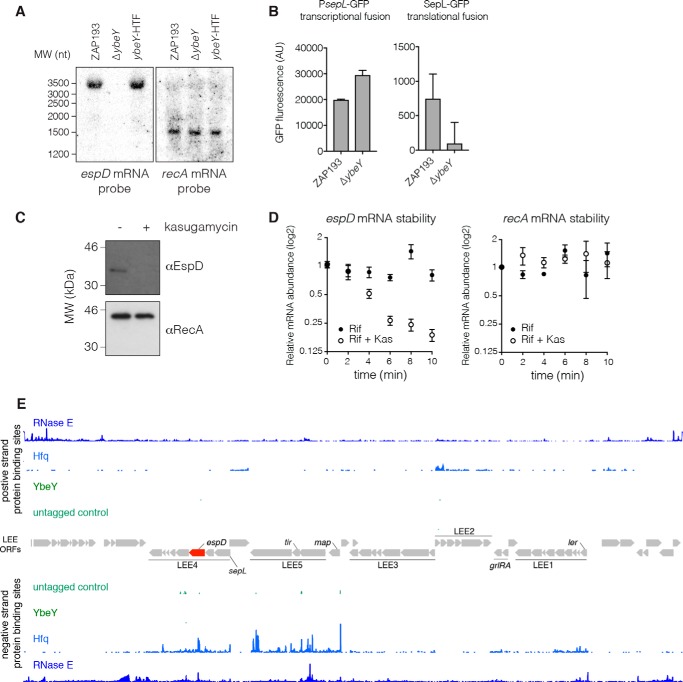
**Translation stabilizes the *espD* transcript.** *A*, Northern blot analysis of *espD* (*left panel*) and *recA* (*right panel*) in WT ZAP193, Δ*ybeY*, and the *ybeY*-HTF strain backgrounds. *B*, transcription and translation of the first gene in the LEE4 polycistronic operon, *sepL*, was measured using GFP fusions. *Left*, the transcriptional *sepL*-GFP fusion (pCDR8) containing the native promoter and 8 nt of the *sepL* 5′-UTR was measured in ZAP193 and Δ*ybeY* backgrounds under T3S-permissive conditions. *Right*, the translational *sepL*-GFP fusion (pDW6) containing the native promoter and entire *sepL* CDS fused to GFP was similarly measured under T3S-permissive conditions. *Error bars* represent S.E. from biological triplicates. *AU*, arbitrary units. *C*, Western blotting for the T3S needle tip protein (EspD) in whole cells grown with and without a subinhibitory concentration of the translation initiation inhibitor kasugamycin (50 μg·ml^−1^). RecA was used as a loading control (*bottom panel*). *D*, RNA stability assay of *espD* and *recA* mRNAs with and without translating ribosomes. Transcription was blocked in EHEC str. ZAP193 by adding 1 mg·ml^−1^ rifampicin (*Rif*) (*closed circles*) or rifampicin and 1 mg·ml^−1^ kasugamycin (*Kas*) (*open circles*) at time 0. The relative abundance of mRNAs was monitored using quantitative RT-PCR. *Error bars* represent S.E. from biological triplicates. *E*, binding profiles for RNase E (*dark blue*), Hfq (*blue*), YbeY (*dark green*), and controls (*green*) are shown for transcripts from the LEE (ORFs are in *gray*; *espD* is highlighted in *red*). Protein binding to positive (*top*) and negative strands (*bottom*) of the transcriptome are shown. The polycistronic LEE1–5 transcripts are indicated *above* and *below* relevant ORFs.

Deletion of *ybeY* leads to depletion of polysomes and allows the accumulation of defective 50S and 30S subunits (28, 30, 44). Translating ribosomes can stabilize mRNAs by protecting from RNase attack, which is commonly initiated by RNase E in *E. coli*. We previously reported that the LEE4 and LEE5 polycistronic transcripts are bound by the small RNA chaperone Hfq and RNase E under T3S-inducing conditions in EHEC (37, 38). We therefore hypothesized that reduced T3S protein expression in the *ybeY* mutant was a consequence of reduced ribosome accumulation and transcript destabilization. In the absence of *ybeY*, there was no detectable *espD* transcript as assessed by Northern blotting (Fig. 6*A*). We next asked whether this was a result of reduced transcription or transcript degradation using GFP fusions to the first gene in the LEE4 polycistronic operon (that starts with *sepL* and includes *espD* (Fig. 6*E*)). Using a transcriptional fusion (to the native promoter and first 8 nt of the *sepL* 5′-UTR), we found that LEE4 is transcribed at higher levels in the *ybeY* mutant (Fig. 6*B*, *left*). In contrast, a fusion that includes the entire *sepL* CDS is repressed in the Δ*ybeY* background (Fig. 6*B*, *right*). Collectively, our results indicate that *LEE4* is highly transcribed in the absence of *ybeY* but rapidly degraded in a manner that depends on features within the translational fusion.

To determine whether translation stabilizes the *espD* transcript, we reduced the abundance of translating ribosomes by growing WT EHEC with a subinhibitory concentration of the antibiotic kasugamycin (50 μg·ml^−1^). Kasugamycin binds the 30S subunit and prevents incorporation of initiator tRNA^fMet^, blocking translation initiation (45, 46). Growing the cells with 50 μg·ml^−1^ kasugamycin did not affect growth of EHEC in MEM-HEPES (data not shown). Expression of the needle tip protein EspD was abolished, whereas expression of a control protein, RecA, was not altered (Fig. 6*C*). This result demonstrates that T3S is selectively inhibited by a mild reduction in translation initiation.

To determine whether *espD* mRNA stability is altered by reduced translation, we determined the lifetime of the LEE4-encoded transcript *espD* after treatment with rifampicin (1 mg·ml^−1^) to inhibit RNA transcription either alone or in combination with kasugamycin (1 mg·ml^−1^) (Fig. 6*D*). In the presence of kasugamycin, the transcript was rapidly degraded relative to the untreated control. Collectively, these results demonstrate that *espD* is sensitive to depletion of initiating ribosomes and rapidly degraded in a *ybeY* mutant or under conditions where translation is limited. Our recent analysis of Hfq- and RNase E–binding sites in EHEC (37, 38) (Fig. 6*E*) indicates that *espD* binds RNase E, and we propose that the polycistronic LEE4 transcript is preferentially degraded by RNase E when mature 70S ribosomes are depleted as in a Δ*ybeY* background or when translation initiation is inhibited by antibiotics.

## Discussion

Here, we report that a screen for loss of T3S in enterohemorrhagic *E. coli* identified the endoribonuclease YbeY. In addition, *ybeY* reduced motility and caused cold sensitivity in EHEC. YbeY has characterized functions in 16S rRNA 3′ cleavage and ribosome quality control (30) and is proposed to regulate sRNA networks (25, 26, 29, 31, 33). Using UV cross-linking and stringent, denaturing purification of YbeY-RNA complexes in EHEC, we found that YbeY binds 16S rRNA *in vivo*. However, we did not recover statistically significant binding sites within sRNAs or mRNAs. Our results are in contrast to recent RNA immunoprecipitation-sequencing (RIP-Seq) analysis of SmYbeY in *S. meliloti* where mRNAs, antisense RNAs, and sRNA were copurified with SmYbeY (33). These results may reflect a functional divergence of YbeY between *S. meliloti* and enteric bacteria. In enteric bacteria, deletion of *ybeY* has consistently been reported to cause a 16S rRNA maturation defect, whereas this was not observed in an *S. meliloti* Δ*ybeY* background (33). Saramago *et al.* (33) also did not recover interactions between SmYbeY and rRNA, consistent with WT 16S rRNA maturation in an *S. meliloti* Δ*ybeY* background. Our results demonstrate that YbeY in EHEC binds rRNA but not sRNA or mRNA, indicating that T3S is activated indirectly through rRNA maturation.

Active translation is known to stabilize mRNAs in both eukaryotes and bacteria (14–17). In *E. coli*, this is thought to occur by steric protection of the mRNA either through elongating ribosomes blocking RNase access to the coding sequence or preventing interactions with the 5′-end–dependent RNase E through occlusion of the mRNA 5′-end (14, 24). We therefore used the antibiotic kasugamycin at subinhibitory concentrations to deplete the cell of initiating ribosomes, analogous to the predicted effect of a *ybeY* deletion. Kasugamycin treatment strongly reduced T3S and destabilized the LEE4-encoded transcript *espD*, indicating that defects in ribosome maturation/availability are sufficient to explain the observed effect on T3S. We suggest that many of the pleiotropic phenotypes observed in *ybeY* mutants may be explained by destabilization of mRNAs when mature 70S ribosomes are depleted or through downstream effects on regulatory networks. Our results are also consistent with previous reports that the phenotype of *ybeY* resembles that of *hfq*. Our earlier work identified Hfq- and RNase E–binding sites in EHEC (37, 38) and found that LEE-encoded transcripts were strongly bound by Hfq under T3S-inducing conditions. A major pathway for Hfq regulation is recruitment of RNase E, resulting in target mRNA degradation. We therefore predict that Hfq-bound mRNAs are particularly sensitive to depletion of initiating and elongating ribosomes via RNase E degradation.

Our UV cross-linking data provide base pair resolution of YbeY contacts within ribosomal RNAs. We found that YbeY contacts 16S rRNA within helix 40 on the neck of the 30S ribosome structure. Nucleotide deletions that indicate sites of direct YbeY-RNA contact place the binding site 41.7 Å from the 3′-end of the 16S structure. Notably, the 16S 3′-end structure lacks the 8 terminal nucleotides, which are presumably flexible (47). The longest axis through YbeY is 47.4 Å (48), indicating that YbeY bound at site 2 is likely to be positioned to cleave between the 16S 3′-end and the internal transcribed spacer. Bacterial two-hybrid analysis of YbeY identified interactions with both the GTPase Era and S11, proteins involved in maturation of the 30S small subunit (42). Cryo-EM of Era-bound 30S subunits places Era at the neck of the 16S rRNA structure (43) between the YbeY-binding site and rRNA 3′-end. Our UV cross-linking results are in excellent agreement with these protein–protein interactions and support the hypothesis that YbeY recognition of the 30S subunit requires both Era and 16S rRNA interactions (42). Era association with the 30S subunit is incompatible with S1 binding and translation, indicating that 3′-end cleavage and Era/YbeY dissociation occur before the 30S subunit becomes translationally competent.

In eukaryotic cells, the 3′-end of 18S rRNA is cleaved by the PIN-domain endoribonuclease Nob1, which is unrelated to YbeY. Strikingly, however, CRAC in *Saccharomyces cerevisiae* (49) mapped Nob1 binding to helix 40 of 18S rRNA with contact-dependent deletions at sites almost identical to YbeY binding in 16S rRNA. This reveals either functional convergence or conservation in the endonuclease-binding sites for small subunit pre-rRNA cleavage.

In *S. cerevisiae*, the final Nob1-dependent cleavage of 18S rRNA is preceded by association with the 60S large subunit and structural rearrangements that bring the 3′-end and internal transcribed spacer into position for Nob1 cleavage (50). Our YbeY UV cross-linking data identified another prominent binding site within helix 33 of 16S rRNA. In the mature 16S rRNA structure, this binding site is located on the beak of the small subunit on the opposite face to the helix 40 binding site. Two possible explanations may account for this binding site. If the pre-30S subunit undergoes substantial structural rearrangements before 3′-end cleavage, the neck and beak binding sites may be in closer proximity and represent a single YbeY-binding site used for 3′-end maturation. YbeY and the exoribonuclease RNase R initiate degradation of mature, defective ribosomes *in vitro* (30). An alternative model would be that beak binding represents YbeY interactions with defective 70S ribosomes.

Recent work has demonstrated that the ketolide antibiotics solithromycin and telithromycin inhibit ribosome maturation (51) and are potent inhibitors of T3S in EHEC (52). Our results suggest that these antibiotics block T3S by depleting mature ribosomes in an analogous manner to the *ybeY* deletion. Notably, deletion of *ybeY* attenuates virulence in both *V. cholerae* and *Y. enterocolitica*, indicating that ribosome availability may play a key role in stabilizing many virulence transcripts in pathogenic bacteria. Subinhibitory concentrations of antibiotics that block ribosome biogenesis may function as selective antivirulence drugs by depleting unstable transcripts of protective ribosomes and may present a novel pathway for blocking bacterial pathogenesis.

## Experimental procedures

### Bacterial strains and media

The bacterial strains used in this study are described in Table S1. Bacteria were cultured in lysogeny broth (LB) or MEM-HEPES (Sigma-Aldrich) supplemented with 0.1% (w/v) glucose and 250 nm Fe(NO_3_)_3_ or M9 medium (Sigma-Aldrich) supplemented with 0.5% (v/v) glycerol and 2 mm MgSO_4_. LB and M9 media were solidified with 1.5% (w/v) agar. When required, antibiotics were added to the media at the following final concentrations: kanamycin, 50 μg·ml^−1^; nalidixic acid, 20 μg·ml^−1^; ampicillin, 100 μg·ml^−1^; chloramphenicol, 50 μg·ml^−1^.

### Transposon mutagenesis

Colonies of *E. coli* O157:H7 str. ZAP193 *lacZYA<*>*tir-lacZ* are blue on M9-glycerol agar plates supplemented with 50 μg·ml^−11^ X-Gal (M9GX). A library of random transposon insertions was produced in this strain using the λpir method (34) with S17–1/pUT-miniTn5Km2 as the donor and ZAP193 *lacZYA<*>*tir-lacZ* as the recipient. Conjugation was carried out on LB agar plates with overnight incubation at 37 °C followed by collection into PBS. Transposon insertions were selected on M9GX plus kanamycin and nalidixic acid (to counterselect the donor strain). Colonies with reduced blue color, indicating decreased LacZ production, were selected. These were checked for absence of the pUT vector by Amp^S^, indicating transposition of miniTn5Km2 into the recipient genome.

### Construction of EHEC deletion and insertion strains

Strains were modified using pTOF24-mediated allelic exchange (53) with pCP20-induced *tetRA* cassette removal where indicated. Plasmids and primer sequences used to construct mutant strains are described in Table S2 and Table S3.

### Preparation of T3S culture supernatant proteins and analysis by SDS-PAGE and Western blotting

Type 3 secretion profiles and Western blotting for RecA and EspD were performed as described previously (52) using antibodies described previously (7). Where indicated, 50 μg·ml^−1^ kasugamycin was added to the supplemented MEM-HEPES before inoculation.

### Measurement of LEE1, LEE4, and LEE5 promoter activity

GFP fusions to *ler* (LEE1), *sepL* (LEE4), and *tir* (LEE5) are listed in Table S2 and were measured in WT and mutant backgrounds as described previously (52).

### Analyses of 16S rRNA maturation defects and espD and recA transcript abundance

For rRNA analysis, total RNA was extracted from cultures grown in LB at 37 °C to an *A*_600_ of 0.6 and then shifted to 45 °C for 1 h. Total RNA was extracted using guanidine thiocyanate (GTC)-phenol extraction (54). Total RNA (1 μg) was treated with glyoxal and separated on a 1.5% BPTE-MetaPhor agarose gel. RNA was stained with SYBR Green II. For detection of *espD* and *recA* transcripts, cultures were grown in supplemented MEM-HEPES to an *A*_600_ of 0.8, and total RNA was extracted using GTC-phenol. Total RNA (4 μg) was treated with glyoxal and separated on a 1.2% BPTE-MetaPhor agarose gel. RNA was transferred to a nylon membrane by capillary transfer and probed using ^32^P-labeled oligonucleotide probes as described previously (37).

### CRAC of YbeY-bound RNAs

UV cross-linking analysis of RNA–protein interactions was performed as defined previously (37). PCR-amplified cDNA libraries were sequenced using an Illumina MiSeq platform. Data analysis was performed using the pyCRAC software package (39) as described in Sy *et al.* (40). To identify statistically significant YbeY-binding sites, pyCalculateFDR was used on mapped reads with the following settings: 100-nt flanking sequence for features (−r, 100), *p* value threshold of 0.05 (−m, 0.05), minimum coverage of 10 reads (−min, 10), and 500 iterations (−iterations, 500). YbeY-binding sites with FDR <0.05, recovered in both HTF-tagged replicates and absent from the untagged control, were retained for further analysis. High-throughput sequencing data sets used in this study were deposited at the Gene Expression Omnibus (GEO) under accession number GSE103774, and previously published Hfq and RNase E data sets are under accession numbers GSE46118 and GSE77463. For comparison of YbeY-HTF and Hfq-HTF purification efficiency, protein was purified from 50 ml of OD 0.8 culture without UV cross-linking and purified over a half-volume (50 μl) of M2 anti-FLAG resin using the CRAC protocol (37). Proteins were eluted from resins by boiling in lithium dodecyl sulfate loading buffer and detected using His_6_ monoclonal HRP-conjugated antibody (Thermo Fisher, MA1-21315-HRP).

### RNA stability measurements

*E. coli* O157:H7 str. ZAP193 was cultured to an OD of 0.8 under T3S-inducing conditions (supplemented MEM-HEPES) and treated with rifampicin (1 mg·ml^−1^) or rifampicin and kasugamycin (1 mg·ml^−1^) for 10 min. Samples (2 ml) were extracted at 2-min intervals and transferred to ice-cold phenol/ethanol mixture (5% phenol, 95% ethanol). Cells were collected by centrifugation (4000 × *g*, 10 mins) and stored at −80 °C. RNA was extracted using a GTC-phenol extraction protocol (54). Total RNA (500 μg) was reverse transcribed using Superscript III (Life Technologies). Transcript abundance was measured by quantitative RT-PCR using KAPA Universal QPCR Master Mix (Sigma Aldrich) on a Rotor-Gene real-time PCR instrument (Corbett). Stability assays were performed in biological triplicate. Error bars represent S.E.

## Author contributions

S. P. M., D. T., D. L. G., and J. J. T. conceptualization; S. P. M. and J. J. T. data curation; S. P. M. and J. J. T. formal analysis; S. P. M., B. M. S., J. L. W., and J. J. T. investigation; S. P. M. and J. J. T. visualization; S. P. M., D. T., D. L. G., and J. J. T. methodology; S. P. M., D. T., D. L. G., and J. J. T. writing-original draft; S. P. M., B. M. S., D. T., D. L. G., and J. J. T. writing-review and editing; D. T., D. L. G., and J. J. T. funding acquisition; D. L. G. and J. J. T. project administration; J. J. T. supervision; J. J. T. validation.

## Supplementary Material

Supporting Information

## Footnotes

3 The abbreviations used are:

EHEC: enterohemorrhagic *E. coli*
T3S: type 3 secretion
sRNA: small RNA
CRAC: UV cross-linking and analysis of cDNAs
LEE: locus of enterocyte effacement
X-Gal: 5-bromo-4-chloro-3-indolyl β-d-galactoside
MEM: minimal essential medium
HTF: His_6_-3xFLAG
nt: nucleotides
str.: strain
GTC: guanidine thiocyanate
FDR: false discovery rate
CDS: coding sequence
BPTE: Bis-Tris PIPES EDTA buffer
Bis-Tris: 2-(bis(2-hydroxyethyl)amino)-2-(hydroxymethyl)propane-1,3-diol.
