# Supplementary material for: Ribosome maturation by the endoribonuclease YbeY stabilizes a type 3 secretion system transcript required for virulence of enterohemorrhagic Escherichia coli
Source: J Biol Chem. 2018 Apr 20;293(23):9006–16. doi: 10.1074/jbc.RA117.000300 (PMC5995498; doi:10.1074/jbc.RA117.000300)
Supplement: Supporting Information [file supp_293_23_9006__index.html]

Ribosome maturation by the endoribonuclease YbeY stabilises a type III secretion system transcript required for virulence of enterohemorrhagic Escherichia coli. — Translation stabilises a type III secretion transcript — Ribosome maturation by the endoribonuclease YbeY stabilizes a type 3 secretion system transcript required for virulence of enterohemorrhagic Escherichia coli — Translation stabilizes a type 3 secretion transcript — Supporting Information 

# Ribosome maturation by the endoribonuclease YbeY stabilizes a type 3 secretion system transcript required for virulence of enterohemorrhagic *Escherichia coli*

## Supporting Information

- Source data - Source files for images
- Supplementary Tables S1-S3 - Supplementary Tables S1-S3
