# Supplementary figures and images for: Ribosome maturation by the endoribonuclease YbeY stabilizes a type 3 secretion system transcript required for virulence of enterohemorrhagic Escherichia coli
Source: J Biol Chem. 2018 Apr 20;293(23):9006–16. doi: 10.1074/jbc.RA117.000300 (PMC5995498; doi:10.1074/jbc.RA117.000300)

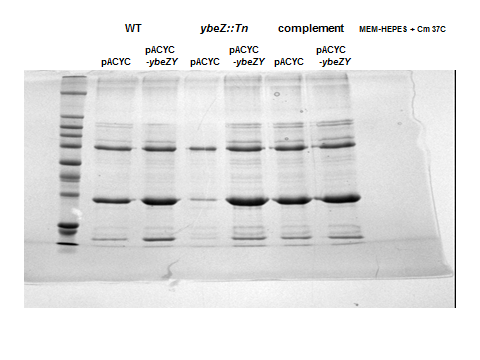

Supplement: Supporting Information [file supp_RA117.000300_132939_2_supp_121337_p7bfyd.zip › source data/Figure 1B source data.tif]

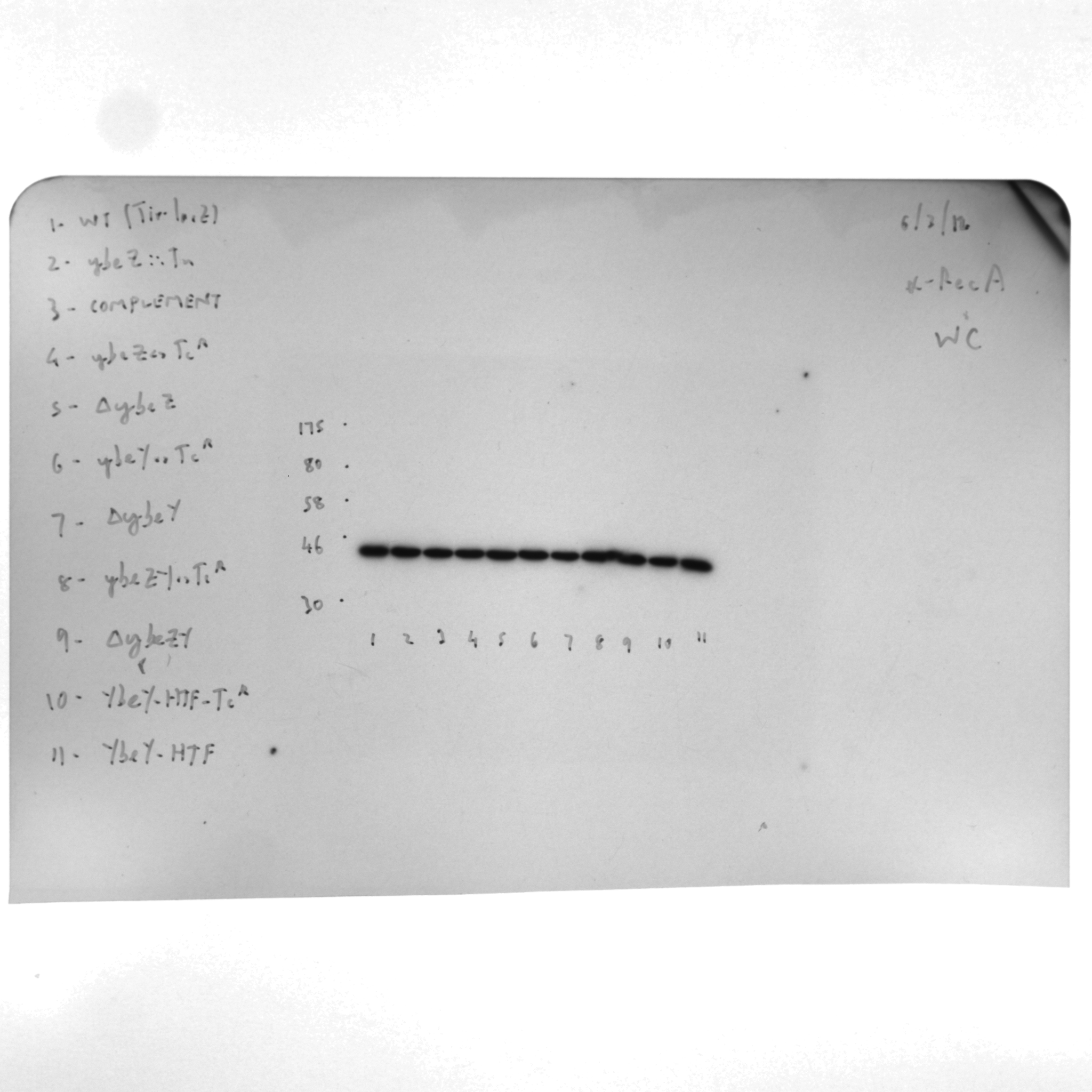

Supplement: Supporting Information [file supp_RA117.000300_132939_2_supp_121337_p7bfyd.zip › source data/Figure 2B bottom source data.tif]

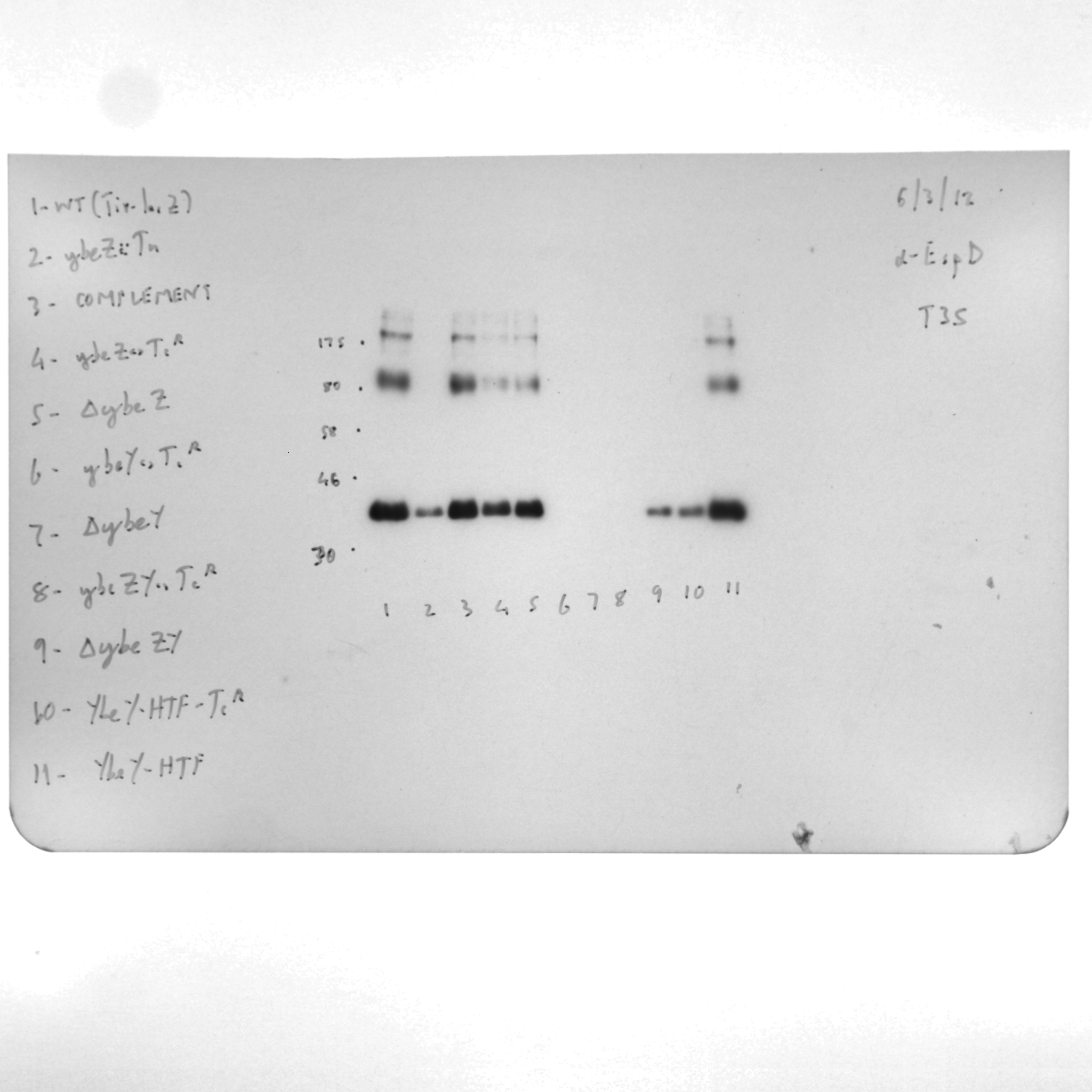

Supplement: Supporting Information [file supp_RA117.000300_132939_2_supp_121337_p7bfyd.zip › source data/Figure 2B top source data.tif]

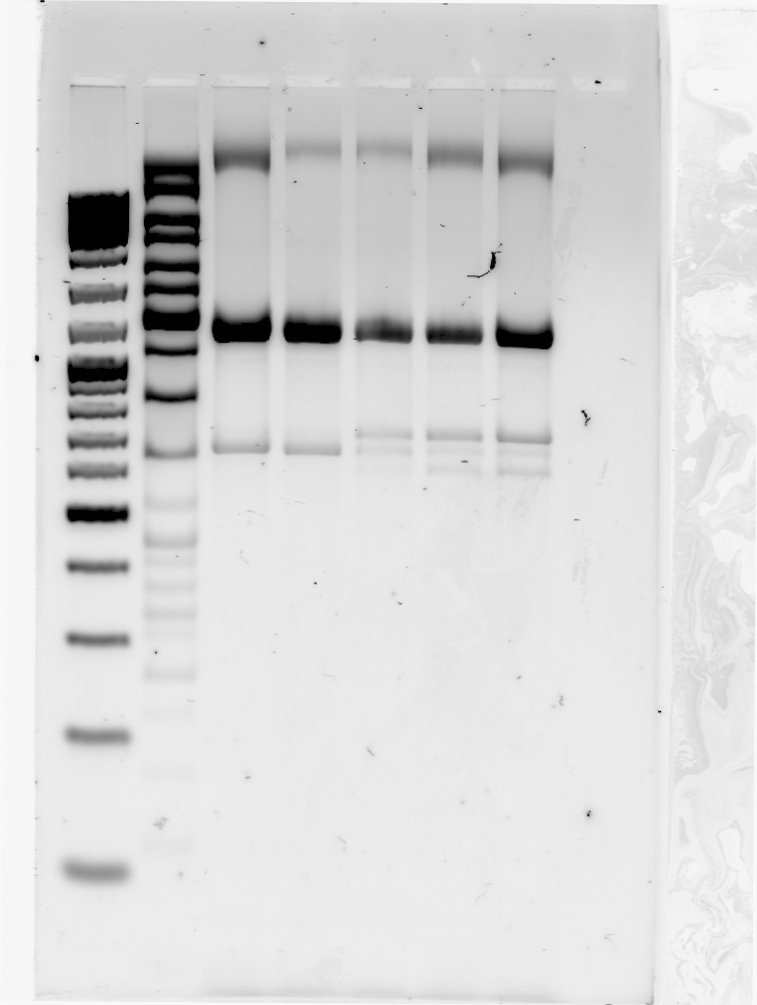

Supplement: Supporting Information [file supp_RA117.000300_132939_2_supp_121337_p7bfyd.zip › source data/Figure 3A source data.tif]

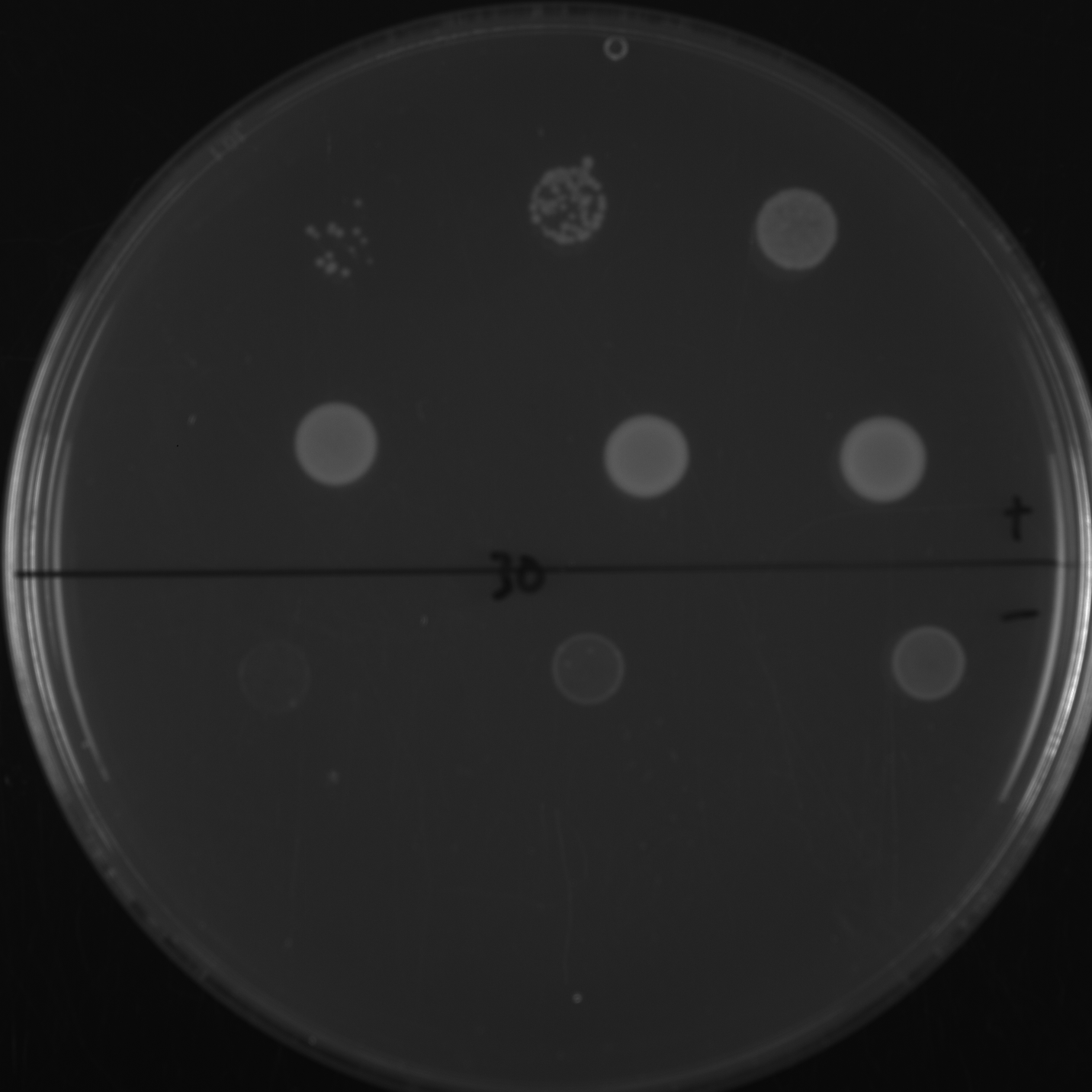

Supplement: Supporting Information [file supp_RA117.000300_132939_2_supp_121337_p7bfyd.zip › source data/Figure 3B source data LB both 30C.tif]

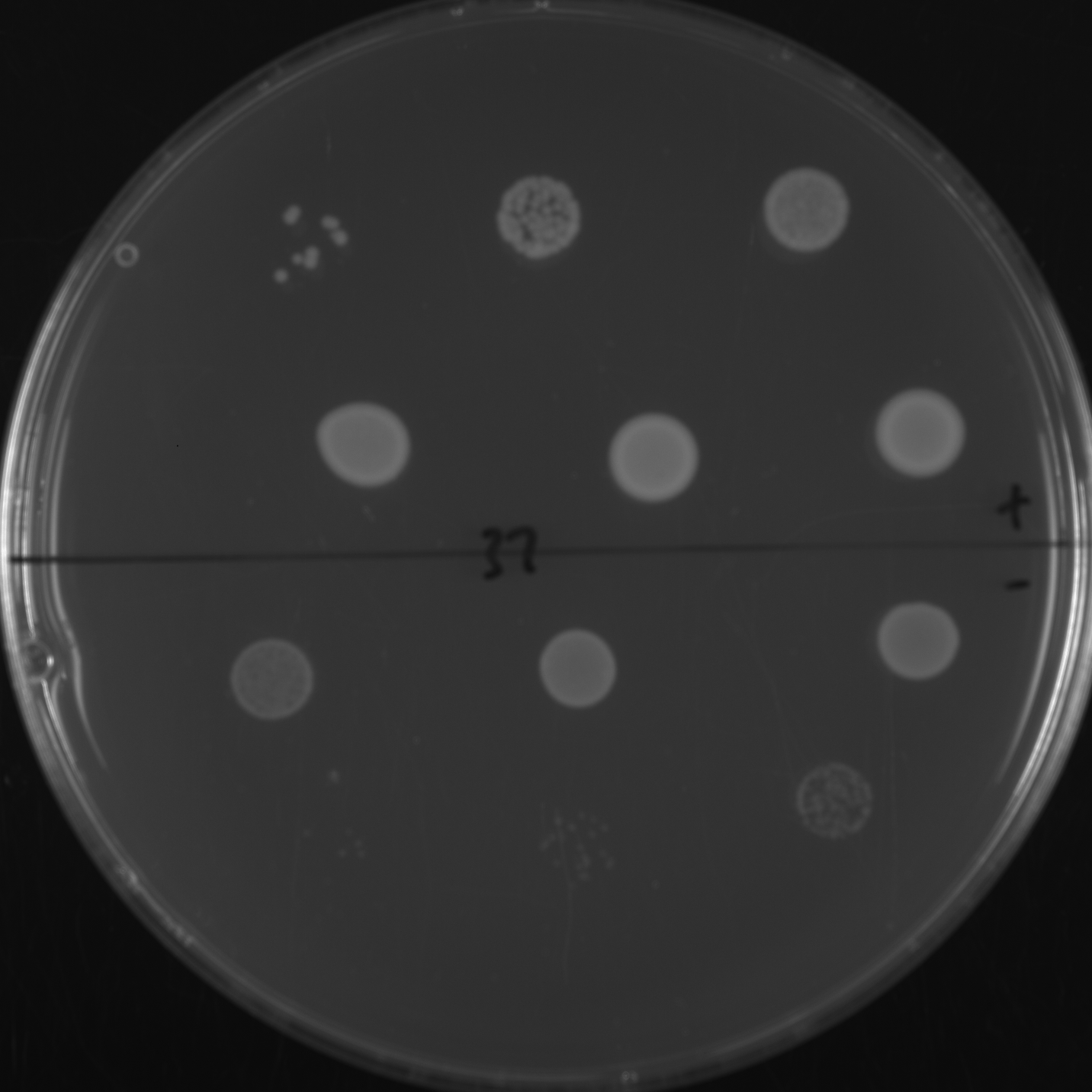

Supplement: Supporting Information [file supp_RA117.000300_132939_2_supp_121337_p7bfyd.zip › source data/Figure 3B source data LB both 37C.tif]

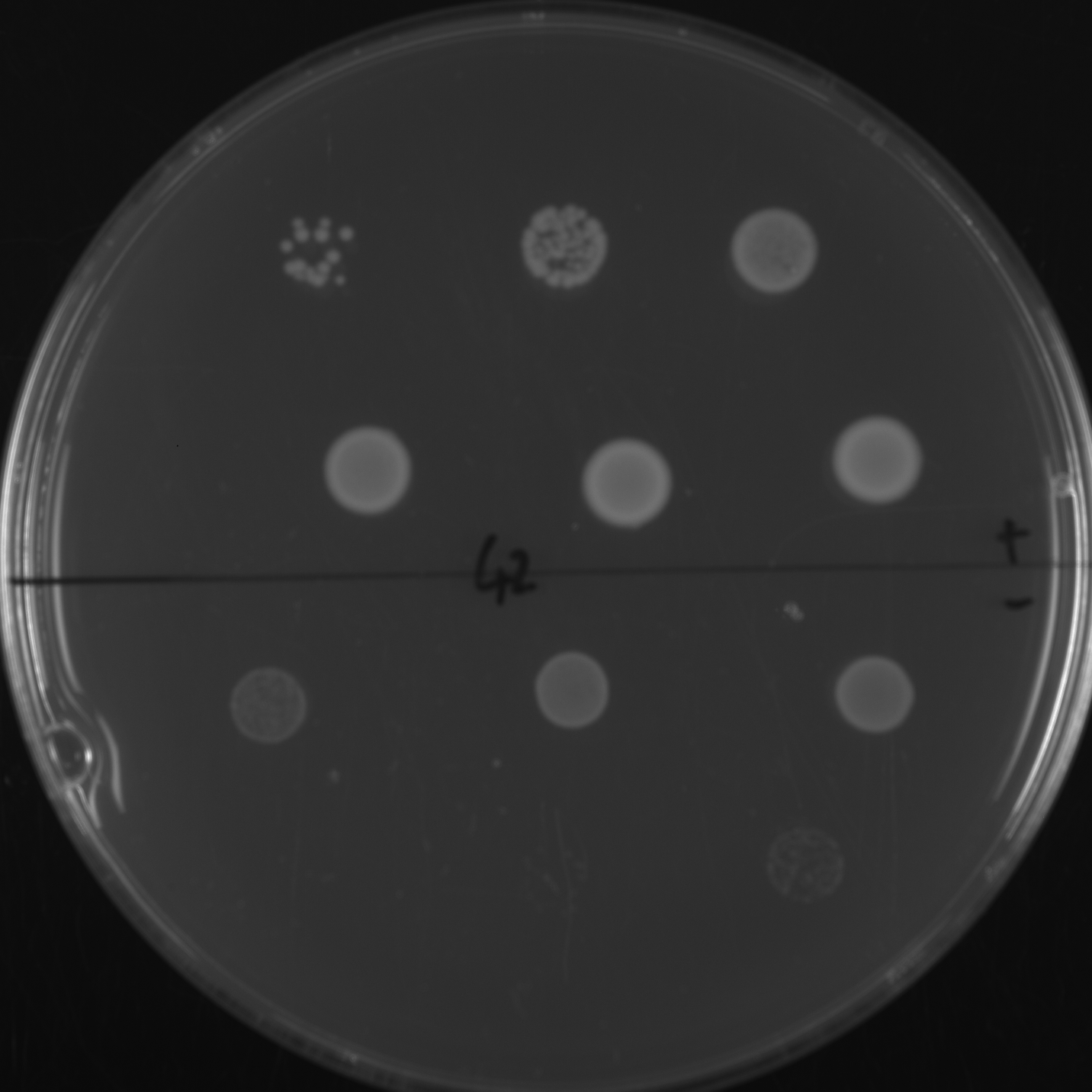

Supplement: Supporting Information [file supp_RA117.000300_132939_2_supp_121337_p7bfyd.zip › source data/Figure 3B source data LB both 42C.tif]

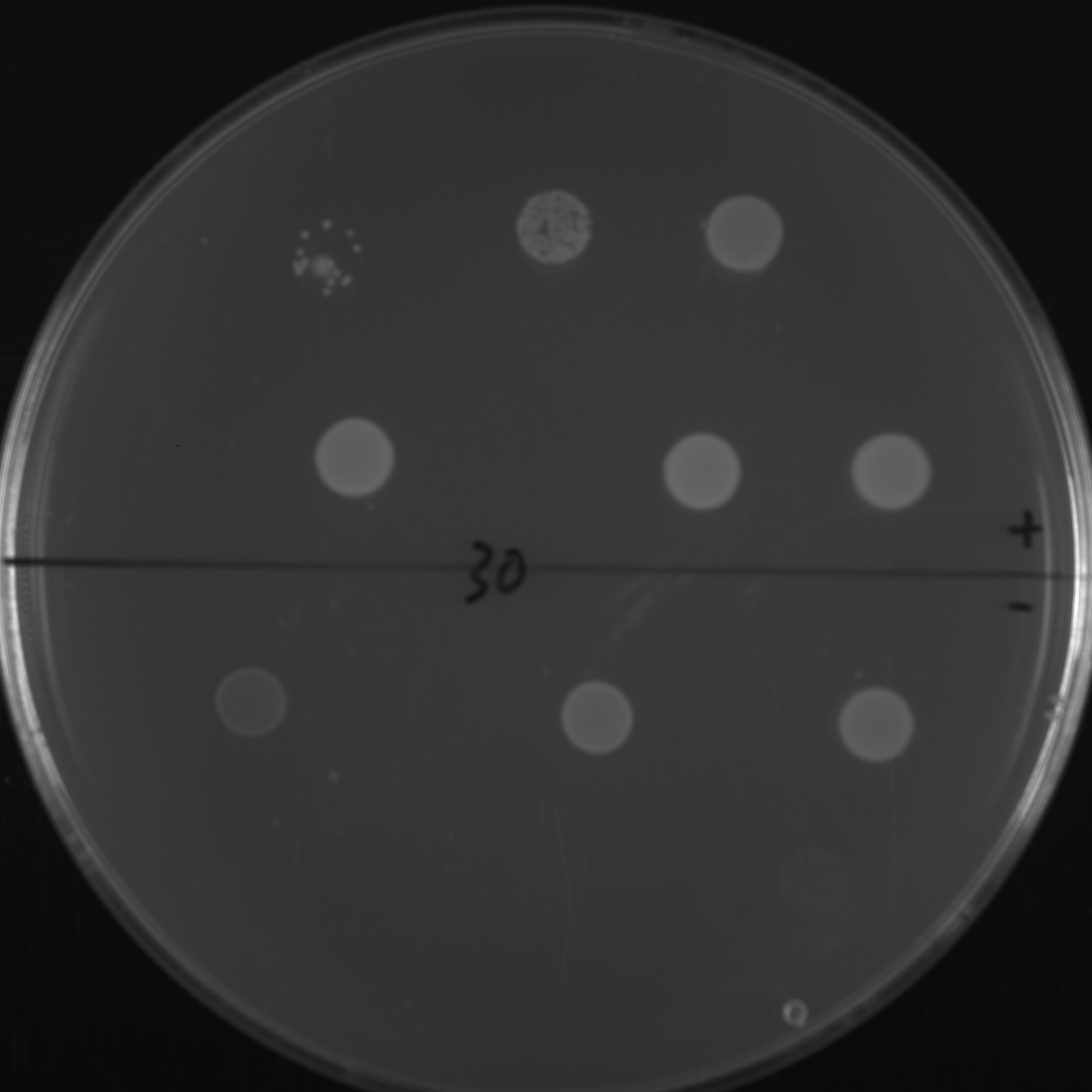

Supplement: Supporting Information [file supp_RA117.000300_132939_2_supp_121337_p7bfyd.zip › source data/Figure 3B source data M9 both 30C.tif]

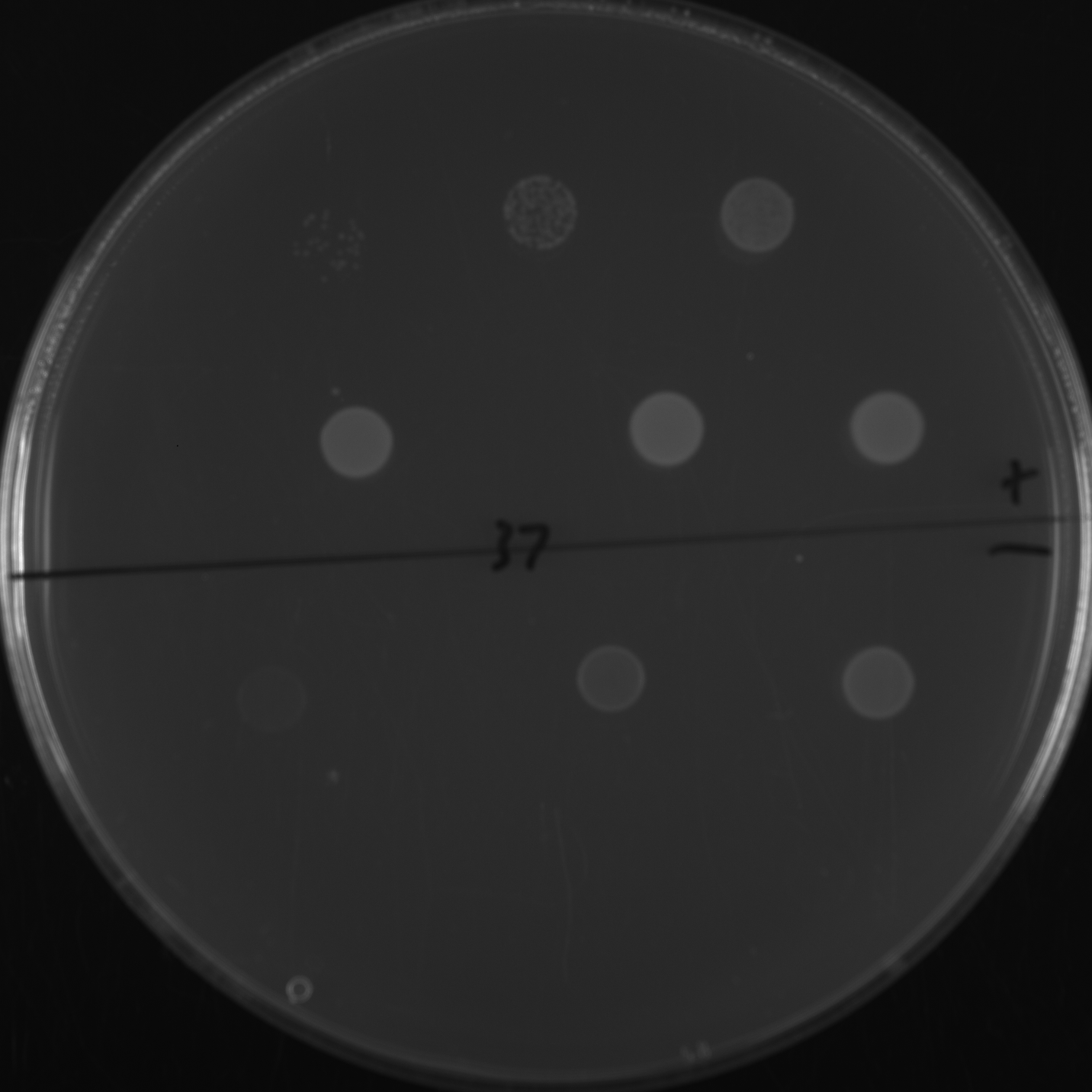

Supplement: Supporting Information [file supp_RA117.000300_132939_2_supp_121337_p7bfyd.zip › source data/Figure 3B source data M9 both 37C.tif]

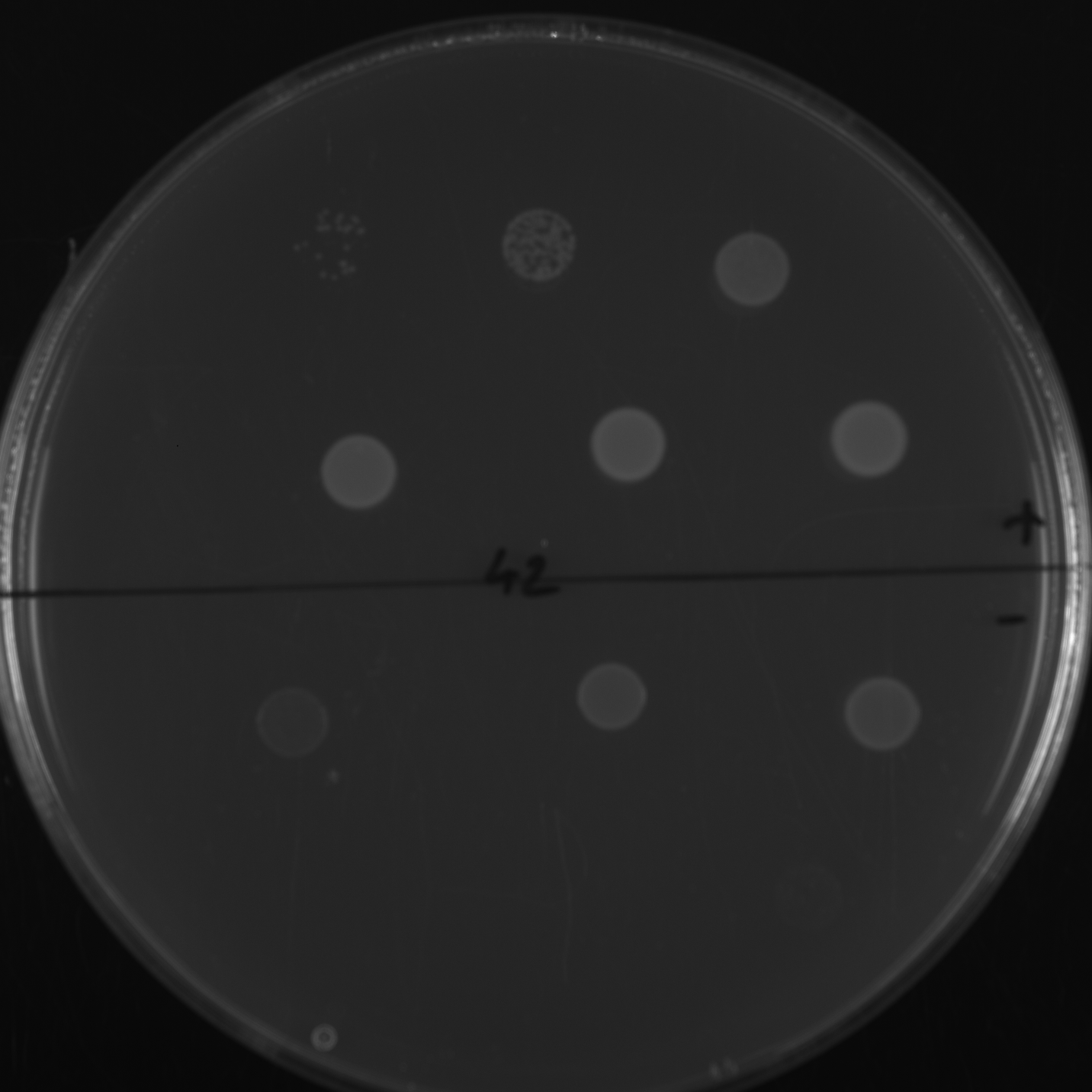

Supplement: Supporting Information [file supp_RA117.000300_132939_2_supp_121337_p7bfyd.zip › source data/Figure 3B source data M9 both 42C.tif]

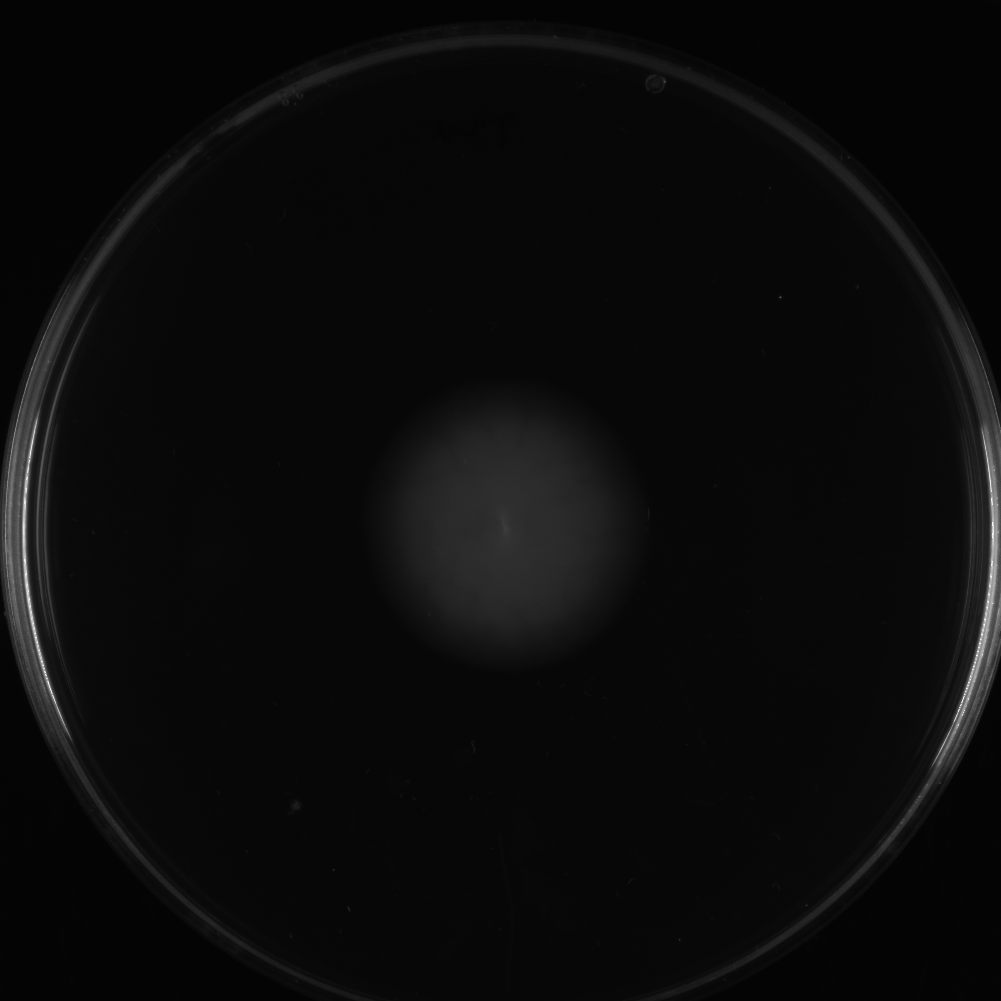

Supplement: Supporting Information [file supp_RA117.000300_132939_2_supp_121337_p7bfyd.zip › source data/Figure 3C source data WT motility LB 37C.tif]

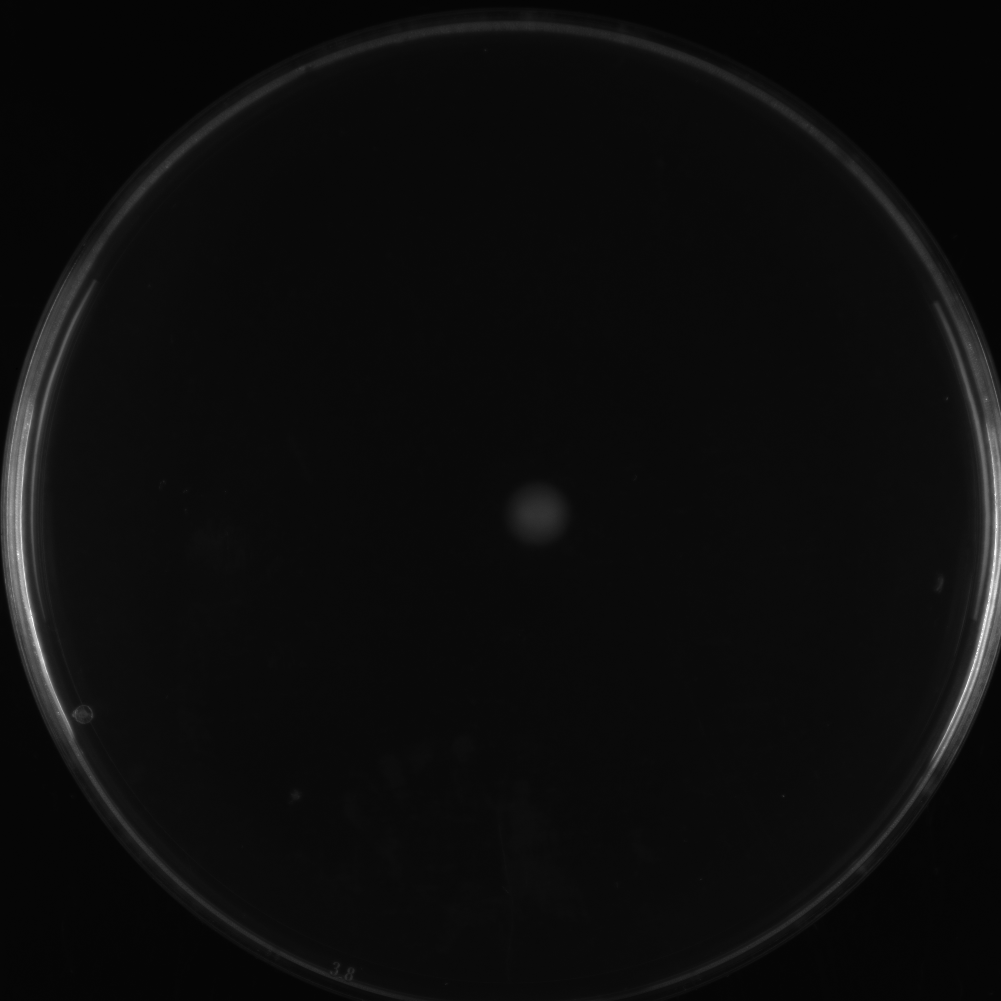

Supplement: Supporting Information [file supp_RA117.000300_132939_2_supp_121337_p7bfyd.zip › source data/Figure 3C source data ybeY-delete motility LB 37C.tif]

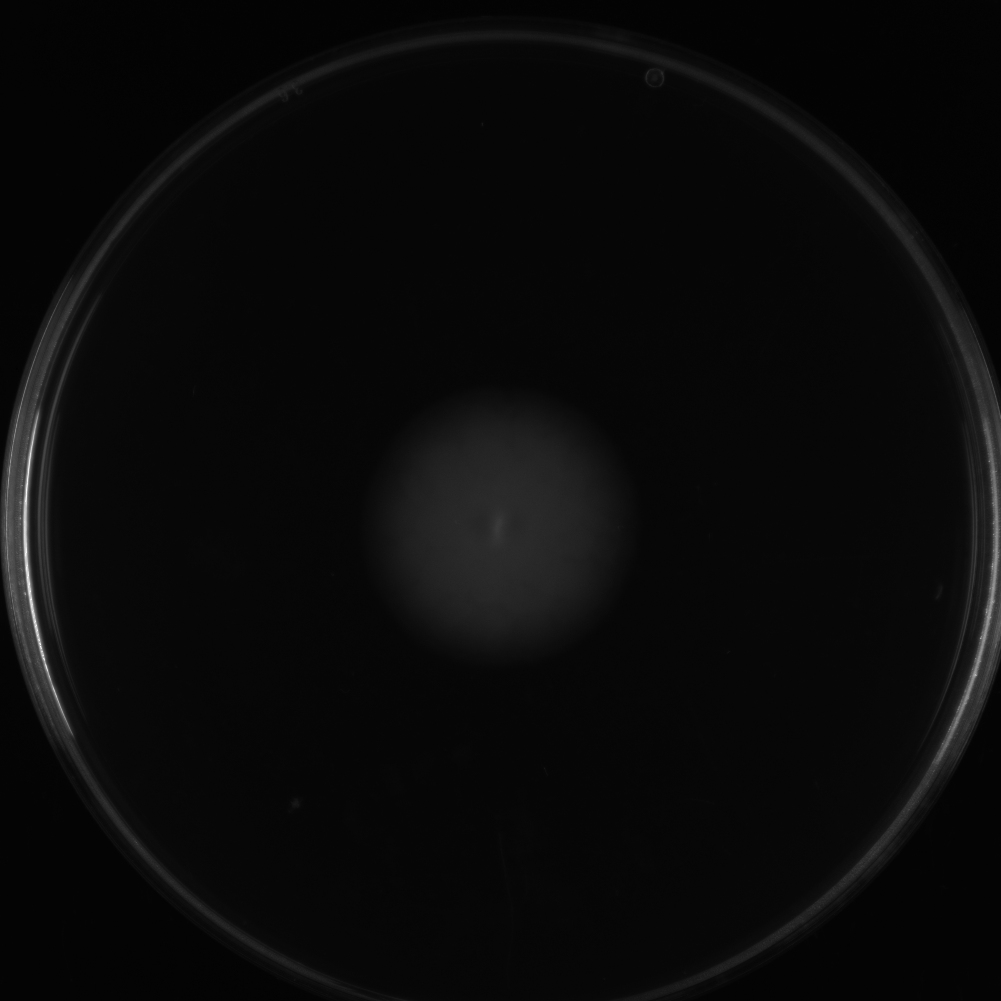

Supplement: Supporting Information [file supp_RA117.000300_132939_2_supp_121337_p7bfyd.zip › source data/Figure 3C source data ybeZ-delete motility LB 37C.tif]

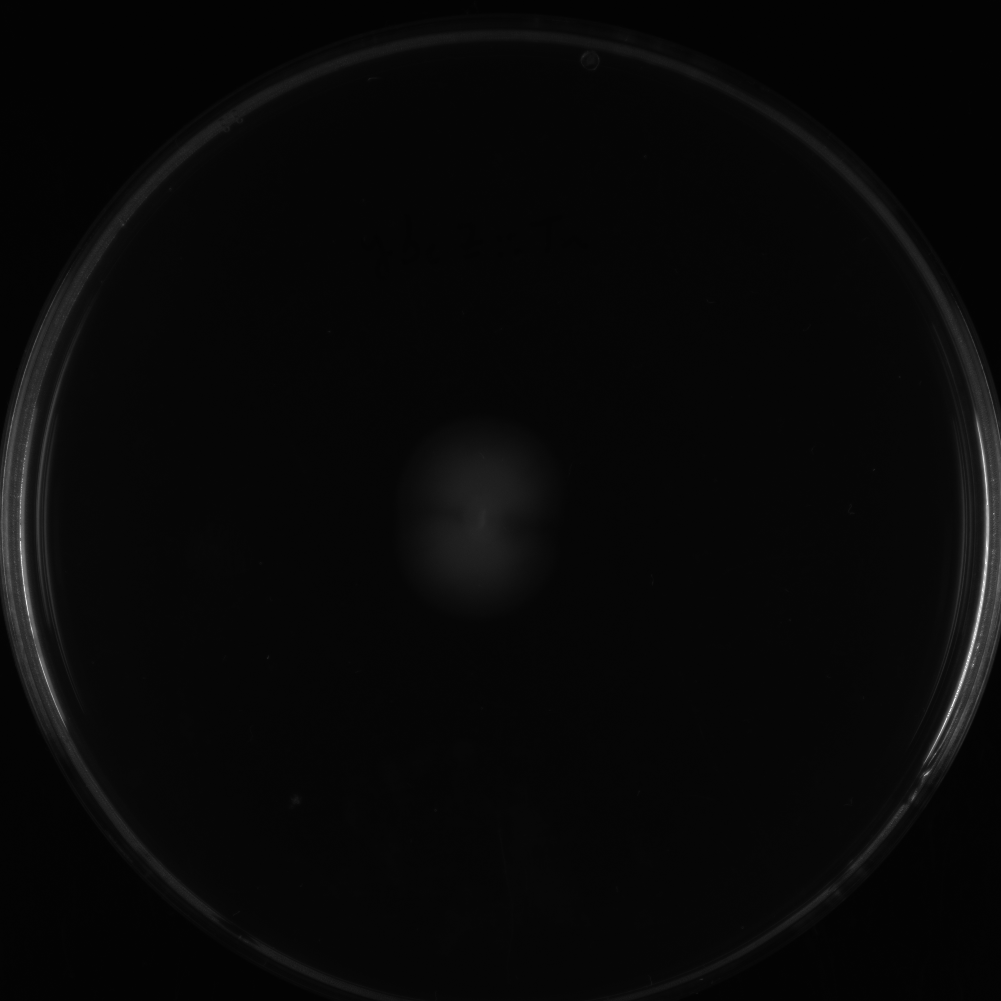

Supplement: Supporting Information [file supp_RA117.000300_132939_2_supp_121337_p7bfyd.zip › source data/Figure 3C source dataybeZ-Tn motility LB 37C.tif]

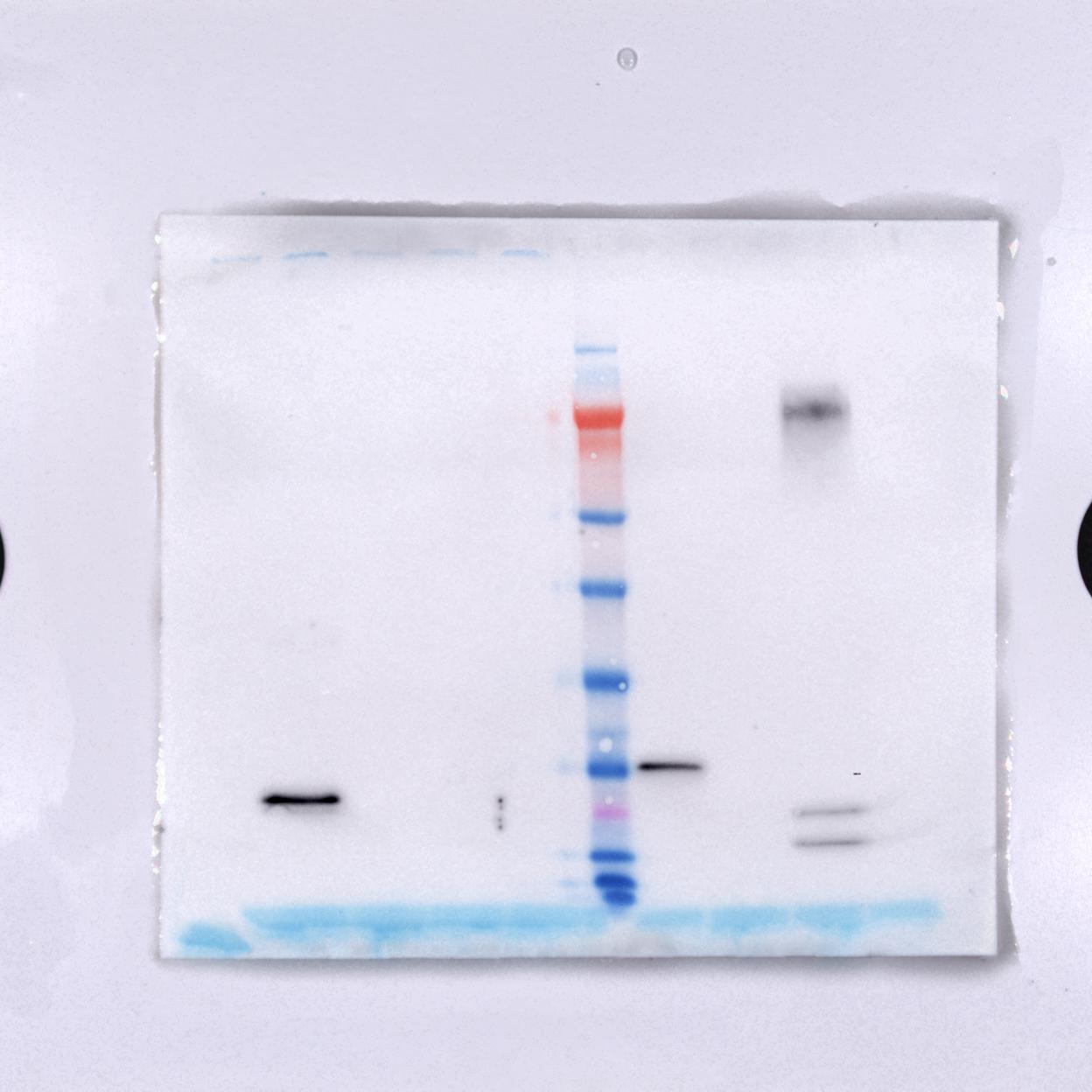

Supplement: Supporting Information [file supp_RA117.000300_132939_2_supp_121337_p7bfyd.zip › source data/Figure 4B source data.jpg]

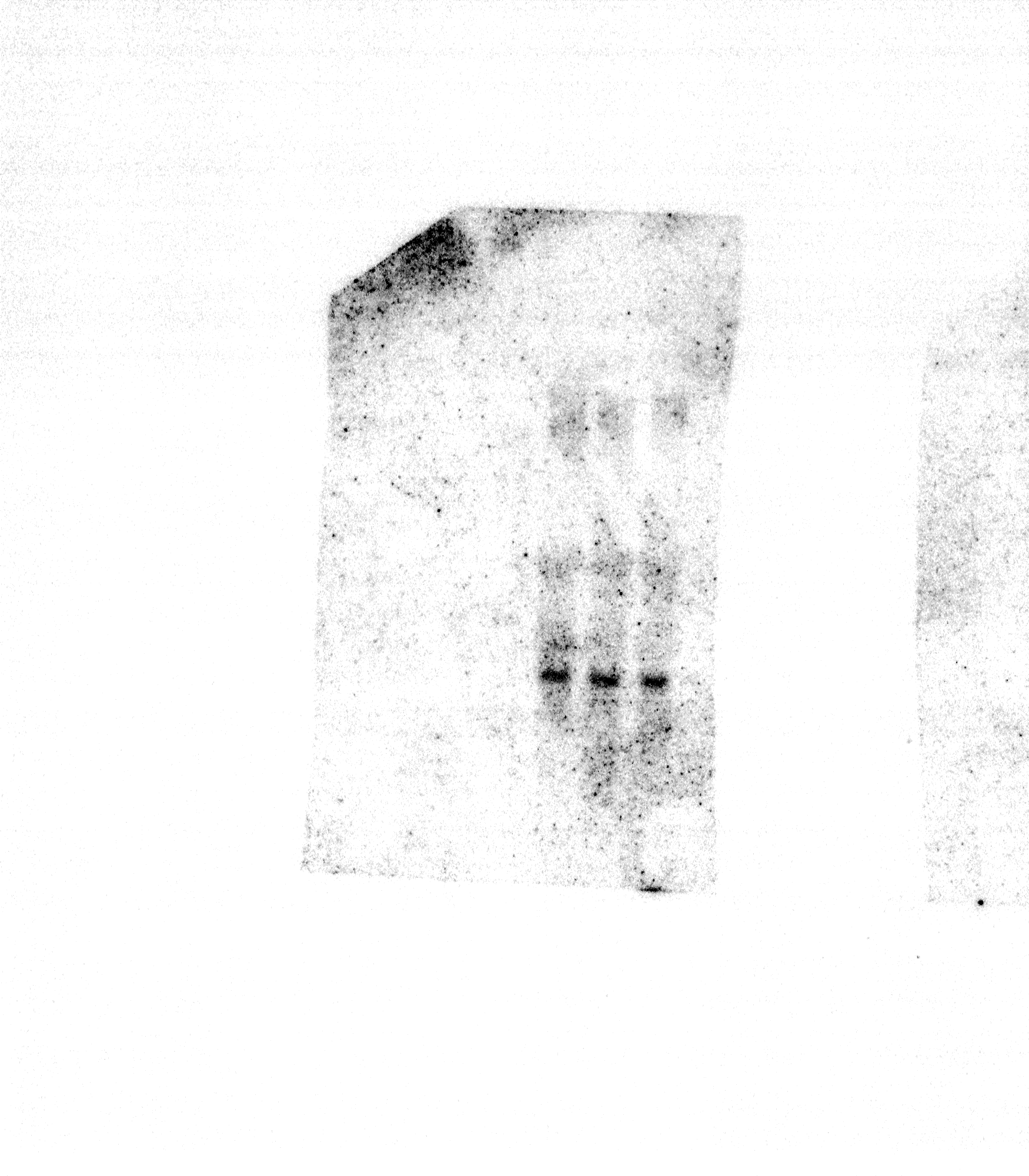

Supplement: Supporting Information [file supp_RA117.000300_132939_2_supp_121337_p7bfyd.zip › source data/Figure 6A bottom source data.tif]

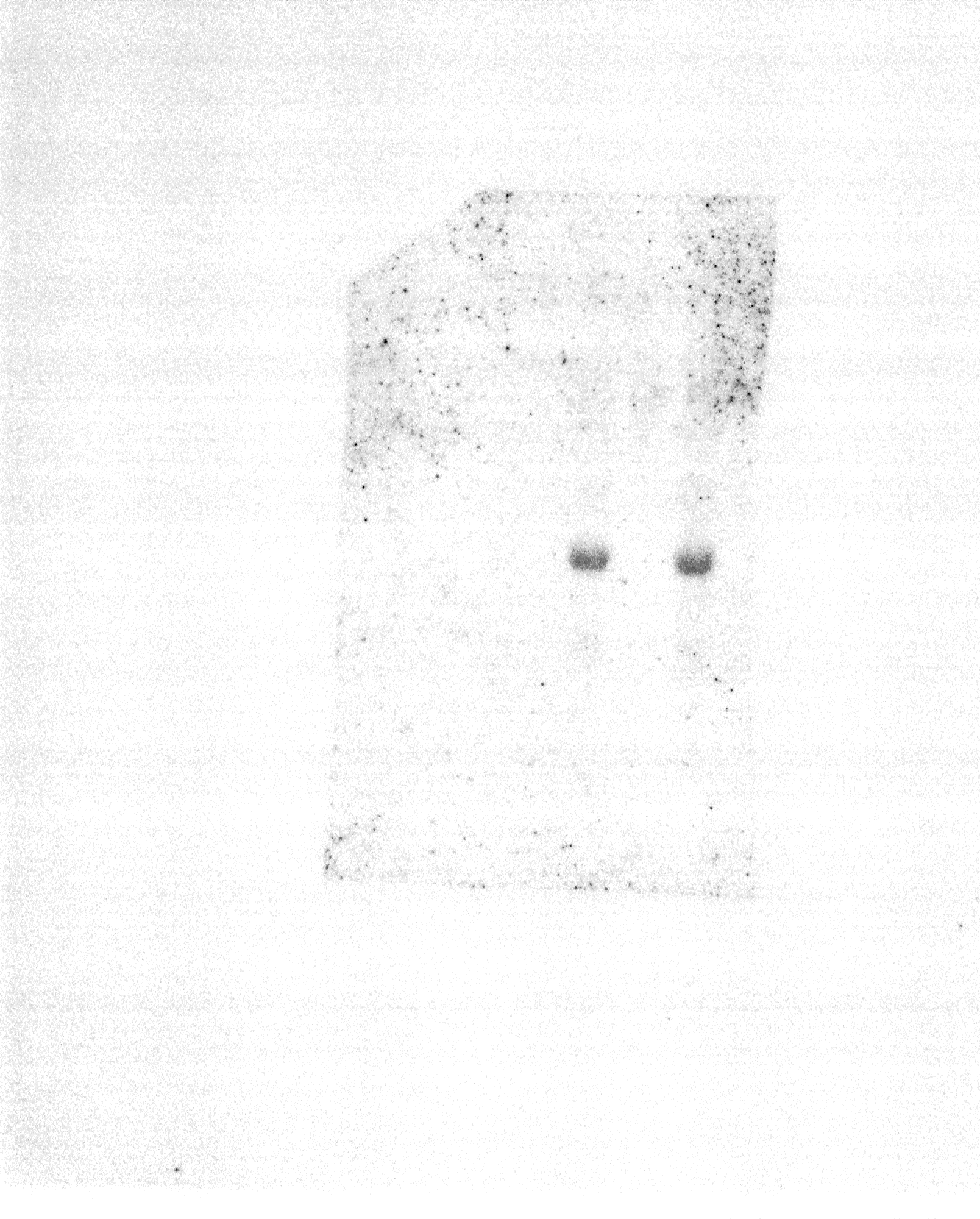

Supplement: Supporting Information [file supp_RA117.000300_132939_2_supp_121337_p7bfyd.zip › source data/Figure 6A top source data.tif]

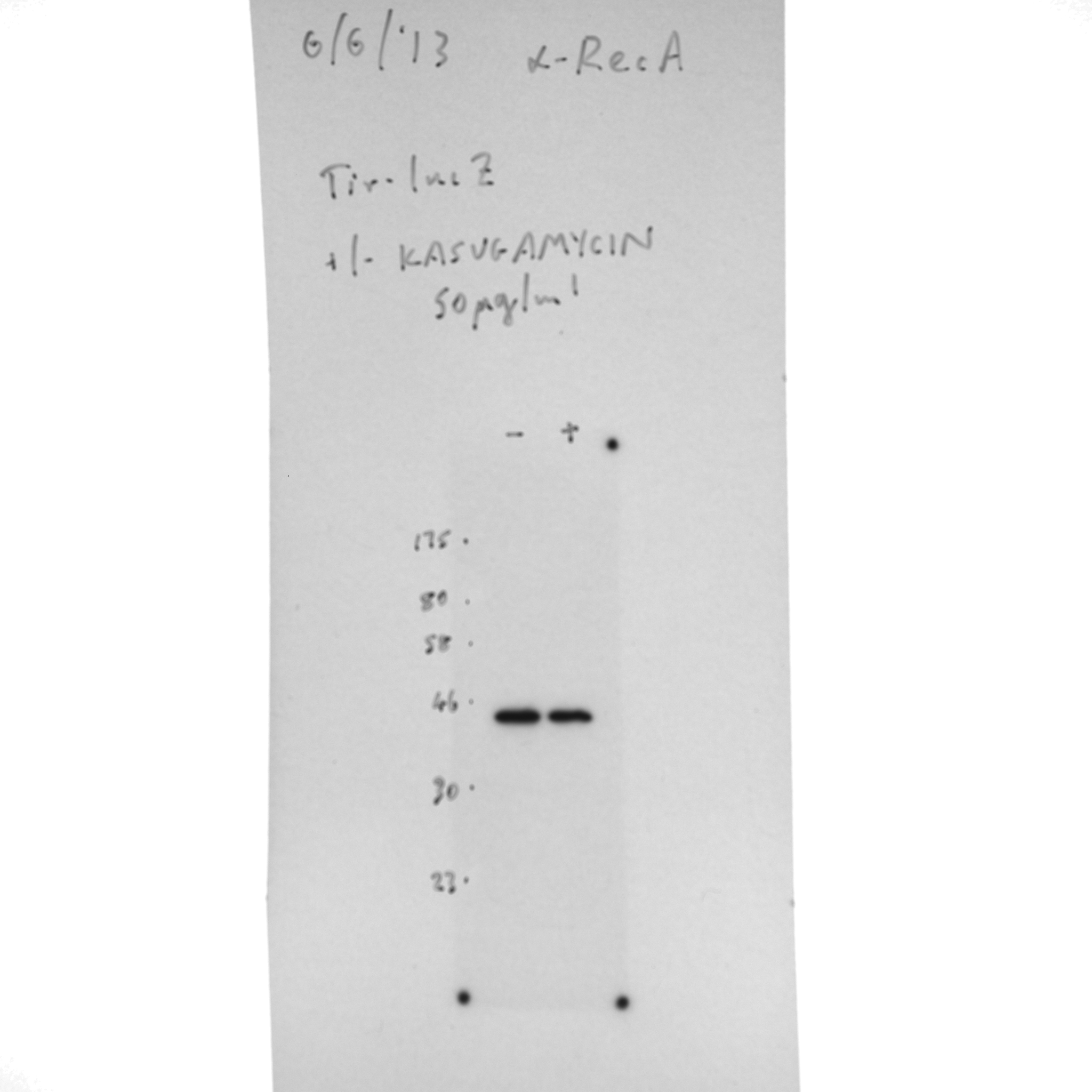

Supplement: Supporting Information [file supp_RA117.000300_132939_2_supp_121337_p7bfyd.zip › source data/Figure 6C bottom source data.tif]

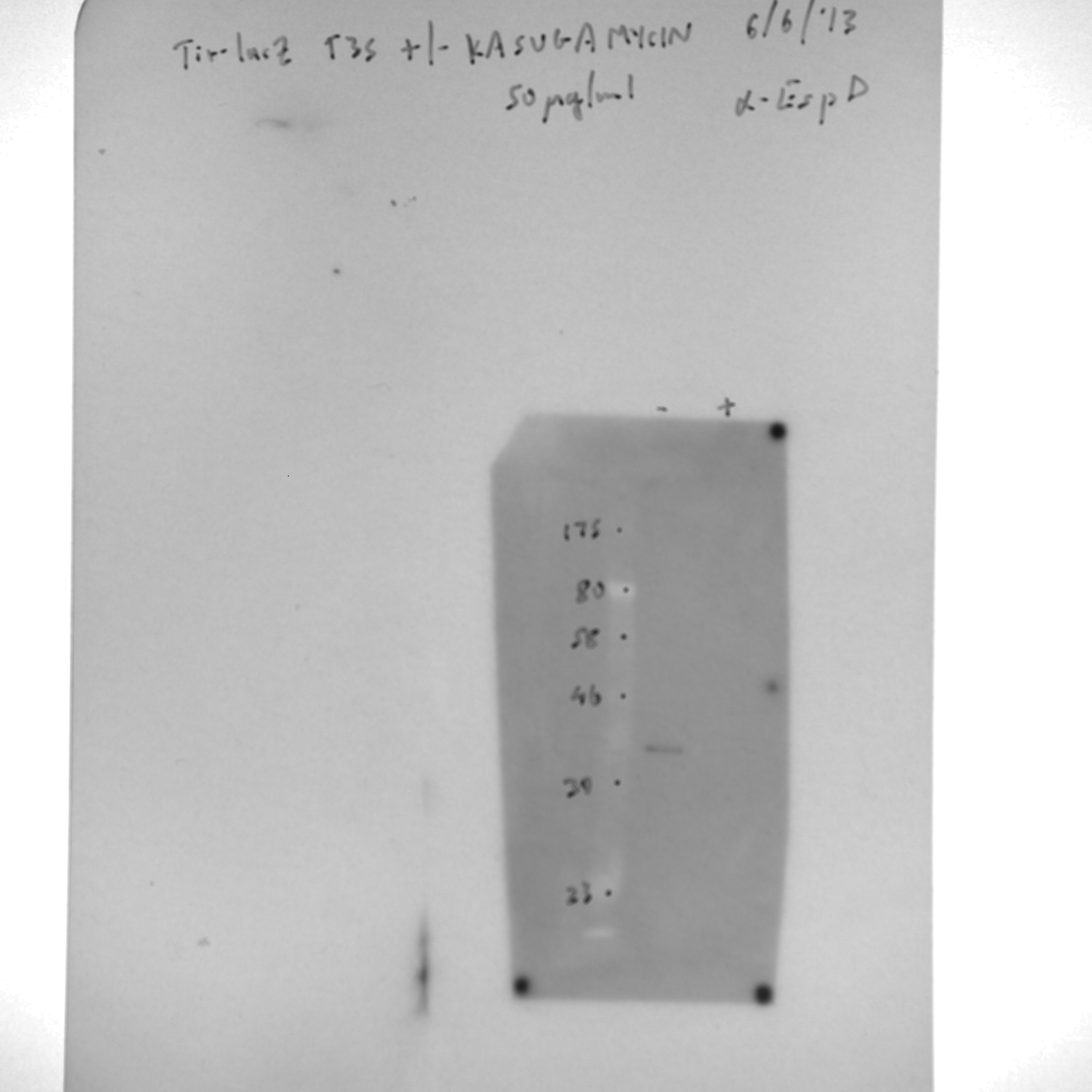

Supplement: Supporting Information [file supp_RA117.000300_132939_2_supp_121337_p7bfyd.zip › source data/Figure 6C top source data.tif]
