# Supplementary material for: Ribosome maturation by the endoribonuclease YbeY stabilizes a type 3 secretion system transcript required for virulence of enterohemorrhagic Escherichia coli
Source: J Biol Chem. 2018 Apr 20;293(23):9006–16. doi: 10.1074/jbc.RA117.000300 (PMC5995498; doi:10.1074/jbc.RA117.000300)
Supplement: Supporting Information [file supp_RA117.000300_132939_2_supp_121338_p7bfyh.pdf]

Supplementary Table S1. Bacterial strains used in this study.

Strain	Description	Source or reference
ZAP193	*E. coli* O157:H7, Stx negative, NCTC 12900, NalR/KanS/AmpS	(Roe et al., 2003)
ZAP193 *lacZYA<>tir-lacZ*	Translational fusion, random transposon mutagenesis recipient	Roe AJ (pers. comm.)
S17-1/pUT-miniTn5Km2	Random transposon mutagenesis donor, KanR/AmpR	(De Lorenzo and Timmis, 1994)
ZAP193 *lacZYA<>tir-lacZ ybeZ::Tn5*	Random transposon mutagenesis exconjugant, NalR/KanR/AmpS	This work
ZAP193 *lacZYA<>tir-lacZ ybeZ::Tn5* rescue	Allelic exchange with pTOF24-*ybeZ*(R), NalR/KanS	This work
ZAP193 *lacZYA<>tir-lacZ ybeZ<>tetRA*	Allelic exchange with pTOF24-*ybeZ*<>tetRA, NalR/TetR	This work
ZAP193 *lacZYA<>tir-lacZ ybeY<>tetRA*	Allelic exchange with pTOF24-*ybeY*<>tetRA, NalR/TetR	This work
ZAP193 *lacZYA<>tir-lacZ ybeZY<>tetRA*	Allelic exchange with pTOF24-*ybeZY*<>tetRA, NalR/TetR	This work
ZAP193 *lacZYA<>tir-lacZ ybeZ*	*tetRA* removal with pCP20, NalR/TetS	This work
ZAP193 *lacZYA<>tir-lacZ* Δ*ybeY*	*tetRA* removal with pCP20, NalR/TetS	This work
ZAP193 *lacZYA<>tir-lacZ* Δ*ybeZY*	*tetRA* removal with pCP20, NalR/TetS	This work
ZAP193 *lacZYA<>tir-lacZ ybeY-HTF-tetRA*	Allelic exchange with pTOF24-*ybeY*-HTF-*tetRA*, NalR/TetR	This work
ZAP193 *lacZYA<>tir-lacZ ybeY-HTF*	*tetRA* removal with pCP20, NalR/TetS	This work

Supplmentary Table S2. Plasmids used in this study.

Plasmid	Description	Source or reference
pTOF24	Allelic exchange vector	(Merlin et al., 2002)
pTOF24-ybeZ(R)	*ybeZ::Tn5* rescue construct	This work
pAJR71	pACYC-LEE1-*gfp*	(Roe et al., 2003)
pAJR75	pACYC-LEE5-*gfp*	(Stevens et al., 2004)
pACYC184	Cloning vector	
pACYC184-ybeZY	Entire operon plus native promoter	This work
pTOF24-ybeZ<>tetRA	*ybeZ* KO construct	This work
pTOF24-ybeY<>tetRA	*ybeY* KO construct	This work
pTOF24-ybeZY<>tetRA	*ybeZY* KO construct	This work
pTOF24-YbeY-HTF-tetRA	*ybeY*-HTF construct	This work
pTOF1-tetRA	Source of *tetRA* cassette	(Tree et al., 2014)
pJET-HTF-tetRA	Source of HTF-*tetRA* cassette	(Tree et al., 2014)
pCP20	*tetRA* cassette removal	(Cherepanov and Wackernagel, 1995)

Supplmentary Table S3. Oligonucleotides used in this study.

Primer	Used to construct plasmid	Sequence
Nt-ybeZ-SalI	pTOF24-*ybeZ*(R)	aaaaa gtcgac cagccgtaattctcaggcccgccg
Ct-ybeZ-PstI	pTOF24-*ybeZ*(R)	aaaaa ctgcag atgcgcccagtgcgcctccagtgg
Nt-ybeZY-XhoI	pACYC184-*ybeZY*	aaaaa ctcgag attctcaggcccgccgttccggtg
Ct-ybeZY-HindIII	pACYC184-*ybeZY*	aaaaa aagctt cgttaatcaccaacggcggggacg
No-ybeZ	pTOF24-*ybeZ*<>tetRA<br>pTOF24-*ybeZY<>tetRA*	aaaaa ctgcag gatatcatcgaagtgtatcgcgac
Ni-ybeZ	pTOF24-*ybeZ<>tetRA*<br>pTOF24-*ybeZY<>tetRA*	cgctcttgcggccgcttggaacgg caccggcctataaggaaattattc
Co-ybeZ	pTOF24-*ybeZ<>tetRA*	aaaaa ctcgag tcagggtaatcatctgggagc
Ci-ybeZ	pTOF24-*ybeZ<>tetRA*	ccgttccaagcggccgcaagagcg aagaacaggaacaaaaatgagtc
No-ybeY	pTOF24-*ybeY<>tetRA*	aaaaa ctgcag atcgaaccggaacagatccacc
Ni-ybeY	pTOF24-*ybeY<>tetRA*	cgctcttgcggccgcttggaacgg gtaaatcgaggatcacctgactc
Co-ybeY	pTOF24-*ybeY<>tetRA*<br>pTOF24-*ybeZY<>tetRA*	aaaaa ctcgag gtcgacttcttcatcgctaaagtg
Ci-ybeY	pTOF24-*ybeY<>tetRA*<br>*pTOF24-ybeZY<>tetRA*	ccgttccaagcggccgcaagagcg tttgccagccatttgactggcag
No-ybeY-HTF	pTOF24-YbeY-HTF-*tetRA*	aaaaa ctgcag cgtacgctgaacgacgcatttatc
Ni-ybeY-HTF	pTOF24-YbeY-HTF-*tetRA*	cgctcttagatctttggaacgg ttctttctcggcaatgtacggatc
Co-ybeY-HTF	pTOF24-YbeY-HTF-*tetRA*	aaaaa ctcgag gtcgacttcttcatcgctaaagtg
Ci-ybeY-HTF	pTOF24-YbeY-HTF-*tetRA*	ccgttccaaagatctaagagcg tttgccagccatttgactggcag
BS_EspD_NB	*espD* northern blot	TTGAGAAACACTTTGTAAATAGCTCGCCTG

	probe	
BS_RecA_NB	*recA* northern blot probe	ATACGGATCTGGTTGATGAAGATCAGCAGC
